# Interactional Endocytosis and Transmembrane Transport Promote Cellular Internalization of Nano‐Delivered RNA Drugs for Efficient Control of Crop Diseases

**DOI:** 10.1111/pbi.70689

**Published:** 2026-05-29

**Authors:** Mei Guan, Chao Xie, Yang Xue, Juan Lu, Qinhong Jiang, Meizhen Yin, Shuo Yan, Jie Shen

**Affiliations:** ^1^ State Key Laboratory of Agricultural and Forestry Biosecurity, MARA Key Lab of Surveillance and Management for Plant Quarantine Pests College of Plant Protection, China Agricultural University Beijing China; ^2^ State Key Laboratory of Chemical Resource Engineering Beijing University of Chemical Technology Beijing China

**Keywords:** *Botrytis cinerea*, cellular internalization, delivery mechanism, dsRNA, endocytosis, nanocarrier, transmembrane transport

## Abstract

*Botrytis cinerea* is a widespread plant pathogenic ascomycete that causes grey mould in over 1400 species and impacts global crop yields. Double‐stranded RNA (dsRNA)‐induced gene silencing is a promising technology for pest control, but efficient delivery remains a major challenge. This work presents a star polycation (SPc)‐based nano‐delivery platform that enhances dsRNA internalization and enables effective control of *B. cinerea* . SPc assembles with dsRNA via electrostatic interactions, hydrogen bonding and Van der Waals forces, forming stable nanoscale complexes. SPc facilitates dsRNA uptake into fungal mycelia by 2.19‐fold, primarily via two activated routes: endocytosis and transmembrane transport, which increase vesicle and particle numbers by 3.74‐ and 1.87‐fold, respectively. Six genes (*pkc*, *ypt10*, *pil1*, *mfs1*, *mfs2* and *mfs3*) in these two routes play crucial roles in the delivery process. RNA interference, chemical inhibition and mutation experiments demonstrate that the two routes interact to optimize the cellular uptake of SPc‐loaded dsRNA. Finally, a high‐efficiency RNA fungicide is developed, comparable to commercial fungicide in protecting cucumber leaves and tomato fruits. This study reveals the synergistic mechanism of nanocarrier‐mediated gene delivery and identifies key genes, supporting the design and application of RNA pesticides.

## 1 Introduction

Plant pathogens and pests are major contributors to the significant losses in crop quality and yield, posing a serious threat to global food security considering the rapid population growth (Savary et al. 2019). Currently, chemical/synthesized pesticides remain the primary effective methods for crop protection, but their unscientific application has led to a series of public concerns such as environmental pollution, induced pest resistance, impairment for non‐targets, etc. (Varah et al. 2019; Fisher et al. 2018). Thus, there is an enormous demand for more selective and eco‐friendly alternatives. RNA interference (RNAi) has been widely applied in the identification and analysis of functional genes, which provides new opportunities for the sustainable development of green pest management (Fire et al. 1998; Hernandez‐Soto and Chacon‐Cerdas 2021; Bramlett et al. 2020). As the third revolution in the pesticide field, RNA pesticides display numerous advantages such as high target specificity, low resistance, easy degradation, etc., and the first RNA pesticide has already received commercial approval (Parker et al. 2019; Yan et al. 2024). However, the efficient translocation of double‐stranded RNA (dsRNA) molecules across the biological barriers such as the cell wall/membrane of pathogens and insect cuticle/peritrophic membrane must be achieved for efficient pest management, which remains a huge challenge for designing/developing applicable RNA pesticides.

Cellular internalization of exogenous substances mainly involves passive transport, active transport and endocytosis (Wu et al. 2023). More specifically, the passive transport does not require additional energy, which depends on the concentration gradient to diffuse. The active transport requires additional energy and specific transporter proteins, including major facilitator superfamily (MFS) transporters, ATP‐binding cassette (ABC)‐type transporters, etc. (Drew et al. 2021; Zhang et al. 2023). The endocytosis pathway includes pinocytosis, phagocytosis and receptor‐mediated endocytosis, the latter of which either clathrin‐dependent or independent endocytosis (Khan and Steeg 2020). For dsRNA internalization, current publications have revealed that the translocation is primarily via two mechanisms, including endocytosis and transmembrane channel‐mediated absorption (Vélez and Fishilevich 2018; Cooper et al. 2018). One is the cellular uptake of dsRNA through clathrin‐dependent or independent endocytosis (Saleh et al. 2006), while the other one entails the uptake of dsRNA facilitated by transmembrane channel protein SID‐1 (Feinberg and Hunter 2003). The dsRNA is usually taken up by fungi via clathrin‐dependent endocytosis. For instance, the uptake of exogenous dsRNA is regulated by clathrin heavy chain (CHC), vacuolar H^+^ ATPase 16 kDa subunit (VATpase), ADP ribolysation factor 72A (Arf72A), etc. in the white mould phytopathogen (Wytinck, Manchur, et al. 2020; Wytinck, Sullivan, et al. 2020). The SID‐1‐like transmembrane channel can facilitate the dsRNA uptake in Colorado potato beetle (Cappelle et al. 2016), but it is not working in migratory locust (Shih et al. 2009), indicating the diverse mechanism among species.

Some types of cationic nanocarriers have become an ideal platform for efficient dsRNA delivery (Chao et al. 2023; Pal et al. 2024; Wang, Li, Ying, et al. 2023; Wang, Li, Zhang, et al. 2023; Wang, Yan, Lan, et al. 2023). Carbon dots (CDs) can activate the clathrin‐dependent endocytosis via up‐regulating the expression of *clathrin*, *filamentous actin* (*F‐actin*), *small GTPase Rab5* (*RAB5*) and *syntaxin of plants61* (*SYP61*), which promotes the dsRNA uptake by *Phytophthora* pathogens (Wang, Li, Ying, et al. 2023; Wang, Li, Zhang, et al. 2023; Wang, Yan, Lan, et al. 2023). Star polycation (SPc) with tertiary amines can also up‐regulate the expression of *chc*, *adaptor protein‐2 sigma subunit* (*AP2S1*) and *ADP‐ribosylation factor 1* (*Afr1*), etc. to activate the clathrin‐dependent endocytosis, which promotes the uptake of exogenous RNA molecules by insect and plant cells (Yang et al. 2022; Ma et al. 2022). The chitosan‐loaded dsRNA can be translocated into cytoplasm via multiple mechanisms, with the clathrin‐dependent endocytosis likely playing key roles than canonical SID‐1/SID‐2 pathway (Lichtenberg et al. 2019). Moreover, fungi lack the SID‐1 protein orthologs, and most studies have indicated that the dsRNA uptake is greatly relied on clathrin‐dependent endocytosis (Wytinck, Manchur, et al. 2020). However, the nanocarrier‐based dsRNA delivery mechanism is complicated in fungi, and the potential functions of other delivery routes are still not clear.

As a destructive plant pathogen, *Botrytis cinerea* can cause grey mould disease in over 1400 crop species, and its limited uptake of dsRNA restricts the RNAi efficiency (Qiao et al. 2023; Shen et al. 2021). The SPc‐mediated dsRNA delivery system has been applied to increase the RNAi efficiency in oomycetes and insects, which has been selected for Research Highlights from China collection by Springer Nature (Yan et al. 2020; Wang, Li, Ying, et al. 2023; Wang, Li, Zhang, et al. 2023; Wang, Yan, Lan, et al. 2023). The current study aims to elucidate the complicated mechanism underlying the SPc‐mediated dsRNA delivery in *B. cinerea* , which is beneficial for constructing a nanoplatform to design/develop applicable RNA fungicides. Here, the SPc can self‐assemble with dsRNA via electrostatic interaction, hydrogen bond and Van der Walls force into nanoscale dsRNA/SPc complexes. The complexation with SPc improves the adhesion performance of dsRNA on *B. cinerea* mycelia, which facilitates the rapid cellular uptake of dsRNA. The SPc primarily activates two routes: endocytosis and transmembrane transport, and six genes play crucial roles in this activation for enhanced cellular internalization of dsRNA. Two routes can interact with each other to optimize the uptake of SPc‐loaded dsRNA, and the suppression of either route inhibits the cellular uptake. Finally, a high‐efficiency RNA fungicide co‐targeting *dcl2* and *iqg1* is developed with the aid of SPc, which can remarkably protect the cucumber leaves and tomato fruits from the infection by *B. cinerea* . This study elucidates the complicated synergistic mechanism of nanocarrier for gene delivery via two interactional routes, which provides an excellent nanoplatform for designing/developing novel high‐efficiency RNA drugs.

## 2 Results and Discussion

### 2.1 Self‐Assembly and Characterization of Nanoscale dsRNA/SPc Complexes

**FIGURE 1:**
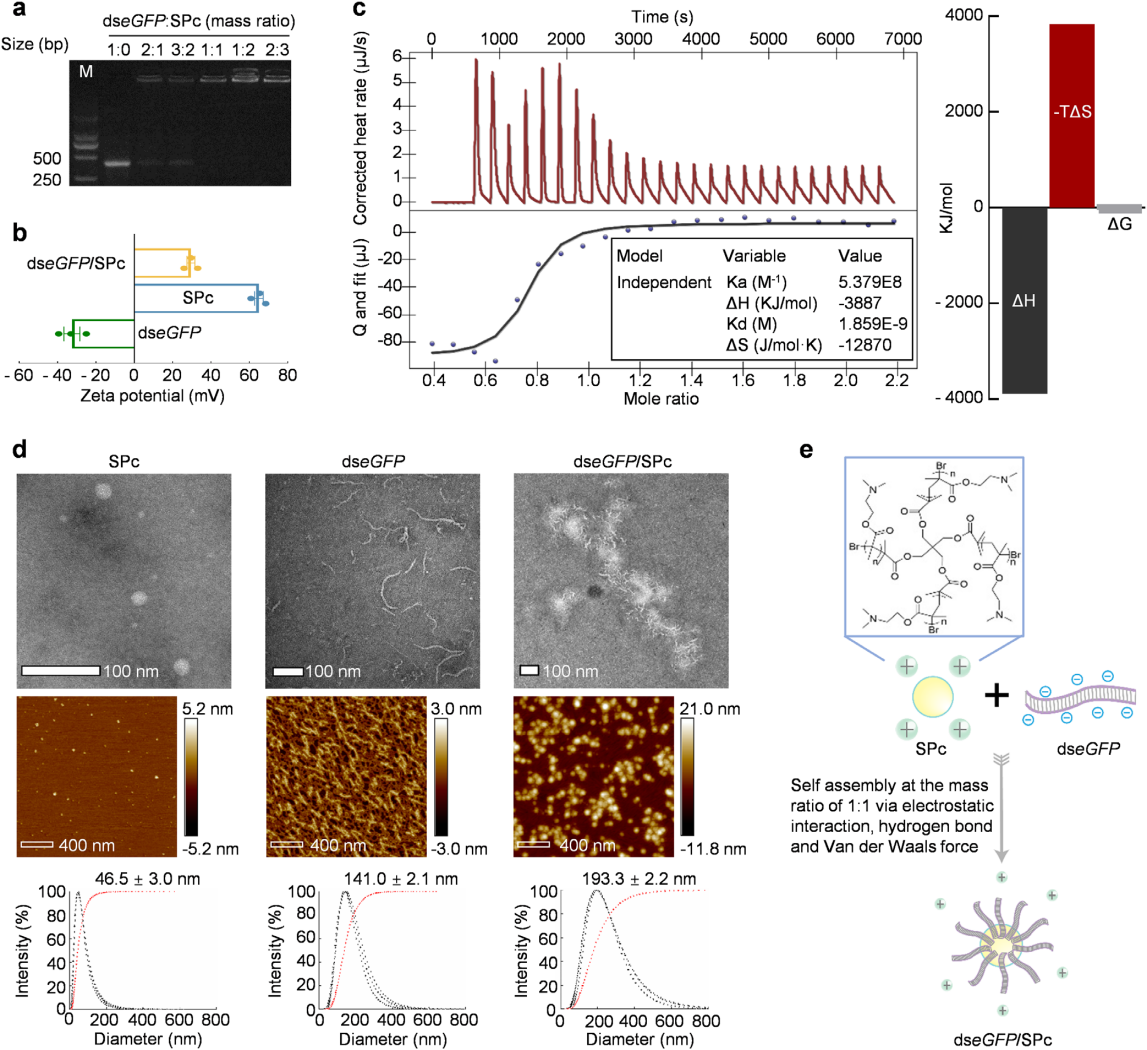
Assembly mechanism and microscopic morphology of SPc/dsRNA complexes. (a) Loading capacity of SPc toward ds*eGFP*. The ds*eGFP* was incubated with SPc at various mass ratios, which was analysed using 2% agarose gel. (b) Zeta potential values of ds*eGFP*, SPc and ds*eGFP*/SPc complex. Each treatment included three independent samples. Bar represents the mean ± SEM. (c) ITC titration of ds*eGFP* (0.0025 mM) into SPc solution (0.00345 mM). The Δ*G* value was calculated using the equation of Δ*G* = Δ*H* − *T*Δ*S*. (d) TEM images, AFM images and particle size distributions of ds*eGFP*, SPc and ds*eGFP*/SPc complex. Each treatment included three independent samples for measuring the particle size distribution. (e) Schematic illustration of self‐assembly process of ds*eGFP*/SPc complexes.

The SPc was successfully synthesized, and its structure has been confirmed by ^1^H NMR (Jiang et al. 2024). The dominant force driving the self‐assembly of dsRNA/SPc complexes was analysed using the gel retardation assay and isothermal titration calorimetry (ITC). As shown in Figure 1a, the band's intensity of the migrated ds*eGFP* (standard sample of dsRNA) gradually decreased with the addition of SPc, and the ds*eGFP* was absolutely trapped at the mass ratio of 1:1. Due to the presence of amine groups (Li et al. 2019), the positively charged SPc (64.94 mV) could bind to the negatively charged ds*eGFP* (−32.51 mV) to reduce the zeta potential to 29.52 mV (Figure 1b), indicating that the electrostatic interaction played an important role in the self‐assembly of ds*eGFP*/SPc complexes. Moreover, the ITC data illustrated that the ds*eGFP* could automatically assemble with SPc due to the negative value of Gibbs free energy change (Δ*G*, −51.74 kJ/mol), and low dissociation constant (*K*_d_) of 1.859 × 10^−9^ M and high affinity constant (*K*_a_) of 5.379 × 10^8^ M^−1^ further indicated that there was an effective and strong interaction between ds*eGFP* and SPc (Figure 1c). The negative values of reaction enthalpy (Δ*H*, −3887 kJ/mol) and entropy change (Δ*S*, −12 870 J/mol·K) suggested that the hydrogen bond and Van der Waals force might be also important for the complexation of ds*eGFP* with SPc. Previous studies have demonstrated that the SPc can act as a universal nanocarrier to assemble with genes/pesticides via various interaction forces, which are mainly dependent on the functional groups of exogenous substances and the spacious structure of SPc (Su et al. 2023; Ma et al. 2022).

Transmission electron microscope (TEM) and atomic force microscope (AFM) were then used to observe the microscopic morphology of ds*eGFP*/SPc complexes (Figure 1d). As depicted, the SPc was quasi‐spherical with lateral size < 100 nm, and the ds*eGFP* was flexural and irregularly linear. The complexation with SPc obviously changed the morphology of ds*eGFP*, and the formed ds*eGFP*/SPc complexes were nearly spherical, indicating that the ds*eGFP* interacted with SPc at multiple sites to result in the blurry and rough surface of SPc. Similar structures have been observed in a previous study that the assembly of DNA with dendritic star polymers forms a dense entwined complex (Yin et al. 2008). In addition, dynamic light scattering (DLS) data revealed that the average sizes of SPc, ds*eGFP* and ds*eGFP*/SPc complex were 46.5, 141.0 and 193.3 nm, respectively. A previous publication has also reported the self‐assembly of dsRNA/SPc complexes, and the particle size increases to 221 nm (Li et al. 2022). The larger particle size of ds*eGFP*/SPc complexes suggested the potential electrostatic adhesion of dsRNA to the positively charged surface of SPc. The self‐assembly mechanism of ds*eGFP*/SPc complexes was summarized in Figure 1e. The advantages of the SPc‐mediated dsRNA delivery system were explored in the following experiment.

### 2.2 Enhanced Stability, Adhesion and Uptake of dsRNA Mediated by SPc

Exogenous dsRNA is highly susceptible to degradation by enzymes in environmental/pest systems, which is a crucial factor to constrain the RNAi efficiency. The incorporation of a nano‐delivery system may be the most efficient method to solve this problem. In the current study, the dsRNA/SPc complexes were incubated with RNase A, and the dsRNA was released by the addition of 0.5% SDS to examine the protective effect of SPc on dsRNA. As shown in Figure S1, the degradation of naked ds*eGFP* (420 bp) or ds*tublin* (1047 bp) occurred quickly after the incubation with RNase A, but no significant difference in the band's density of SPc‐loaded dsRNA treated with RNase A was observed, indicating that SPc could protect short/long dsRNA from degradation to increase stability. Additionally, the band corresponding to SPc‐loaded dsRNA treated with SDS and RNase A showed degradation with a lower molecular weight. This result might be due to the depolymerization of dsRNA detached from SPc branches by SDS, allowing it to be degraded by RNase A. In recent years, various types of nanocarriers have been employed to increase the stability of dsRNA. For instance, the self‐assembly with CDs can protect the dsRNA from degradation by RNase A in the environment (Wang, Li, Ying, et al. 2023; Wang, Li, Zhang, et al. 2023; Wang, Yan, Lan, et al. 2023). Chitosan and dsRNA can be assembled into positively charged nanocomplexes, and its stability is stronger than that of naked dsRNA in hemolymph (Zhou, Jiang, et al. 2023; Zhou, Wang, et al. 2023). The above results suggested that the adhesion of dsRNA to the positively charged surface of SPc might reduce the exposed acting sites for degradation by RNase A.

**FIGURE 2:**
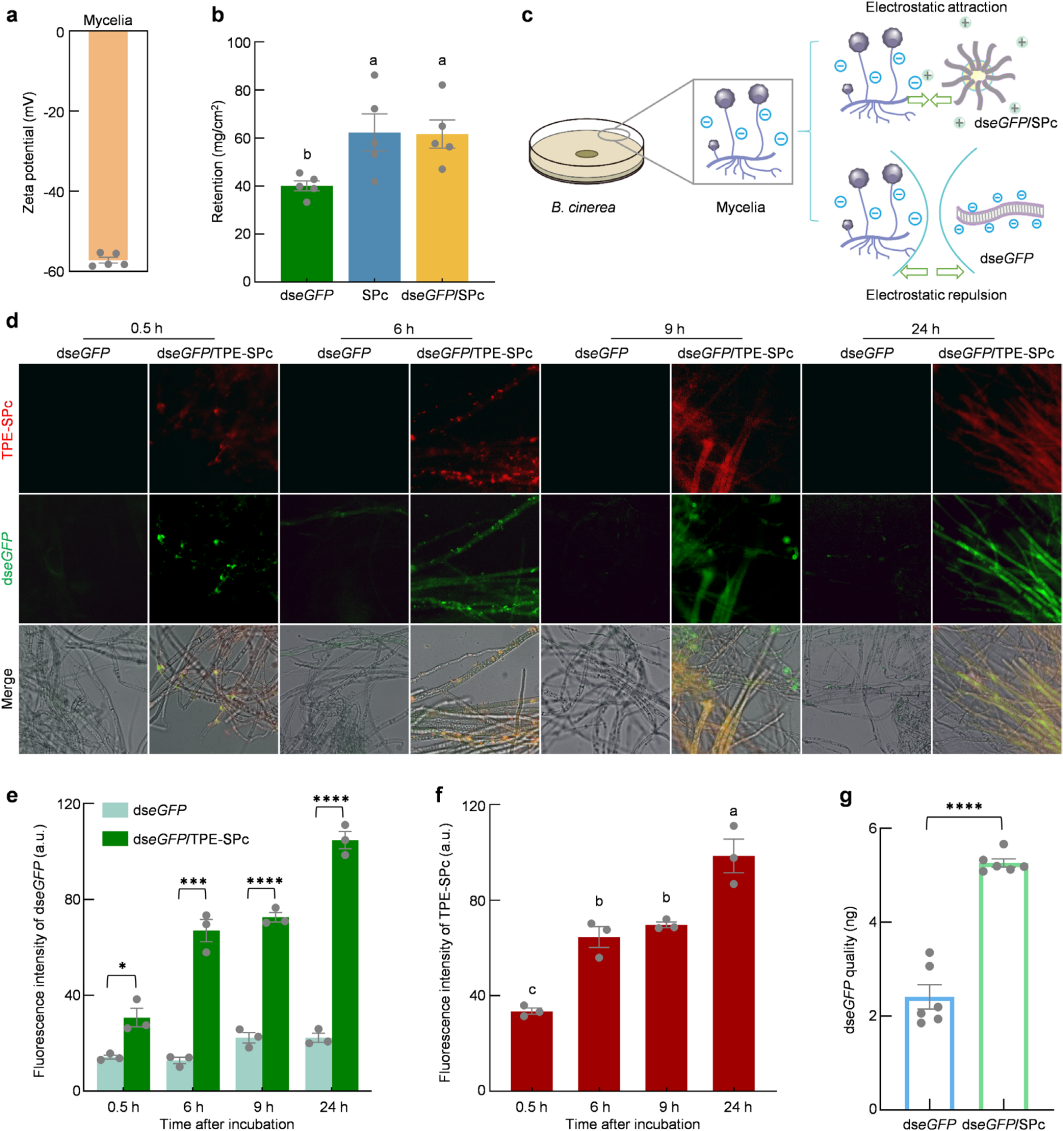
Excellent adhesion performance and uptake of SPc‐loaded dsRNA by *Botrytis cinerea* . (a) Zeta potential value of mycelia (*n* = 5 replicates). (b) Retentions of various solutions on the mycelia discs (*n* = 5 replicates). Different letters above each bar indicate significant differences at *p* < 0.05 as determined by one‐way ANOVA with Tukey HSD test (*F*_2,12_ = 4.89, *p* = 0.0279). (c) Mechanism diagram for increased retention of ds*eGFP*/SPc complexes on mycelia discs. (d) Fluorescence images of mycelia treated with ds*eGFP* and ds*eGFP*/TPE‐SPc complex at different time points. (e) Green fluorescence intensity of mycelia incubated with ds*eGFP*/TPE‐SPc complexes for 0.5, 6, 9 and 24 h (*n* = 3 replications). Fluorescence intensity was quantified by measuring the fluorescence pixel intensity using ImageJ 1.8 software. a.u., arbitrary units. The asterisk indicates significant difference according to independent *t*‐test (two‐tailed, *p < 0.05, ***p < 0.001, ****p < 0.0001). 0.5 h: (*t* = 4.18, df = 4, *p* = 0.0138), 6 h: (*t* = 11.17, df = 4, *p* = 0.0004), 9 h: (*t* = 17.00, df = 4, *p* < 0.0001) and 24 h: (*t* = 20.18, df = 4, *p* < 0.0001). (f) Red fluorescence intensity of mycelia incubated with ds*eGFP*/TPE‐SPc complexes for 0.5, 6, 9 and 24 h (*n* = 3 replications). Fluorescence intensity was quantified by measuring the fluorescence pixel intensity using ImageJ 1.8 software. a.u., arbitrary units. Different letters above each bar indicate significant differences at *p* < 0.05 as determined by one‐way ANOVA with Tukey HSD test (*F*_3,8_ = 39.47, *p* < 0.0001). (g) Quality of ds*eGFP* in the mycelia incubated with naked ds*eGFP* and ds*eGFP*/SPc complex (*n* = 6 replications). The asterisk indicates significant difference according to independent *t* test (Two‐tailed, *t* = 10.42, df = 10, ****p < 0.0001). (a, b, e–g) Bar represents mean ± SEM.

Efficient dsRNA translocation across cell wall/membrane is critical for cross‐kingdom RNAi efficacy, and the delivery efficiency of SPc‐loaded dsRNA was examined. As shown in Figure 2a,b, the retention of positively charged ds*eGFP*/SPc complexes (61.66 mg/cm^2^) on *B. cinerea* mycelia was higher than that of naked ds*eGFP* (40.06 mg/cm^2^) due to the negatively charged surface of mycelia (−57.16 mV). The same surface charge of ds*eGFP* and mycelia caused the electrostatic repulsion, indicating the important role of electrostatic attraction in higher retention of ds*eGFP*/SPc complexes (Figure 2c). This hypothesis is consistent with a previous publication that positively charged surface of nanoparticles aids the interactions with negatively charged cell membranes (Vishwakarma et al. 2024). For instance, cationic polymers are easy to enrich at the edge of bacterial membranes (Sahariah et al. 2023). In addition, positively charged chitosan can penetrate the cell membrane faster than electrically‐neutral and negative chitosan (Yue et al. 2011). The stronger adhesion of SPc‐loaded ds*eGFP* might facilitate its translocation across cell wall/membrane to achieve efficient delivery.

The fluorescent ds*eGFP* was synthesized to incubate with the modified fluorescent SPc (TPE‐SPc) that could be detected at 365 nm. The fluorescent ds*eGFP*/TPE‐SPc complexes were incubated with *B. cinerea* mycelia, and the delivery efficiency was then examined intensively. The fluorescence intensity of ds*eGFP*/TPE‐SPc complexes was consistently higher than that of naked ds*eGFP* at all tested time points (Figure 2d). Furthermore, the absence of an external fluorescent signal was due to the low concentration of ds*eGFP* in the solution. More specifically, the slight fluorescent signal could be detected in the mycelia at 0.5 h after the incubation with ds*eGFP*/TPE‐SPc complexes, which primarily concentrated at the edges of mycelia, appearing as aggregated large particles along with fainter small particles. The fluorescent signal predominantly localized at the centre of mycelia at 6 h, with some aggregated large particles still present at the edges. The punctate fluorescence signal of the ds*eGFP*/TPE‐SPc complex at the hyphal membrane vanished after 9 h, indicating the potential diffusion due to internalization of the complex via endocytosis or active transport within the hyphae. With increasing incubation duration, the fluorescent signal progressively intensified, ultimately permeating all cell wall/membrane, while punctate fluorescent signals at the edges disappeared. The fluorescence intensity of ds*eGFP* increased from 14.21 to 30.73 a.u. and from 22.27 to 104.8 a.u. in the mycelia at 0.5 and 24 h after the incubation with the aid of SPc (Figure 2e). The red fluorescence from TPE‐SPc and green fluorescence from ds*eGFP* completely overlapped, suggesting that the TPE‐SPc facilitated the uptake of ds*eGFP* by mycelia (Figure 2f). The ds*eGFP* content in the mycelia at 6 h after the incubation was quantified using the quantitative real‐time PCR (qRT‐PCR) according to the standard curve (Figure S2). Our results revealed that the ds*eGFP* quality increased from 2.41 to 5.27 ng in the mycelia with the aid of SPc (Figure 2g). Previous studies have also demonstrated that the assembly with SPc can promote the uptake of nucleic acids by insect and plant cells (Yang et al. 2022; Ma et al. 2022). In addition, it has been reported that the zoospores are unable to directly absorb exogenous dsRNA, but the SPc‐loaded dsRNA can penetrate the cell wall/membrane of *Phytophthora infestans* , facilitating its uptake by both spores and mycelia (Qiao et al. 2021; Wang, Li, Ying, et al. 2023; Wang, Li, Zhang, et al. 2023; Wang, Yan, Lan, et al. 2023). Based on above results, the ds*eGFP*/SPc complexes could accumulate on the surface of biological membranes and successfully overcome the delivery barrier, thereby enhancing the delivery efficiency. However, the molecular mechanism underlying the enhanced delivery was still not clear.

### 2.3 Improved Delivery of SPc‐Loaded dsRNA via Activating Endocytosis and Transmembrane Transport

To investigate the potential molecular mechanism in SPc‐mediated dsRNA delivery, the RNA‐seq analysis was performed using the *B. cinerea* mycelia treated with ddH_2_O, SPc, ds*eGFP* and ds*eGFP*/SPc complex. The RNA‐seq results demonstrated high quality and strong correlation, supporting the suitability for subsequent analysis (Table S1 and Figure S3). Venn diagram showed that a total of 250 overlapping DEGs were discovered (Figure S4a). Kyoto Encyclopaedia of Genes and Genomes (KEGG) analysis revealed that the DEGs were enriched in endocytosis, ABC transporters, meiosis, various amino acid metabolisms, transport processes, carbohydrate metabolism, wax biosynthesis, etc. (Figure S4b). More specifically, the incubation with SPc or the ds*eGFP*/SPc complex resulted in a significant up‐regulation of endocytosis‐related genes, including *pkc* and *pil*. Additionally, the genes associated with transmembrane transport (*mfs2*, *mfs3* and *mfs4*) were also activated (Figure S4c).

**FIGURE 3:**
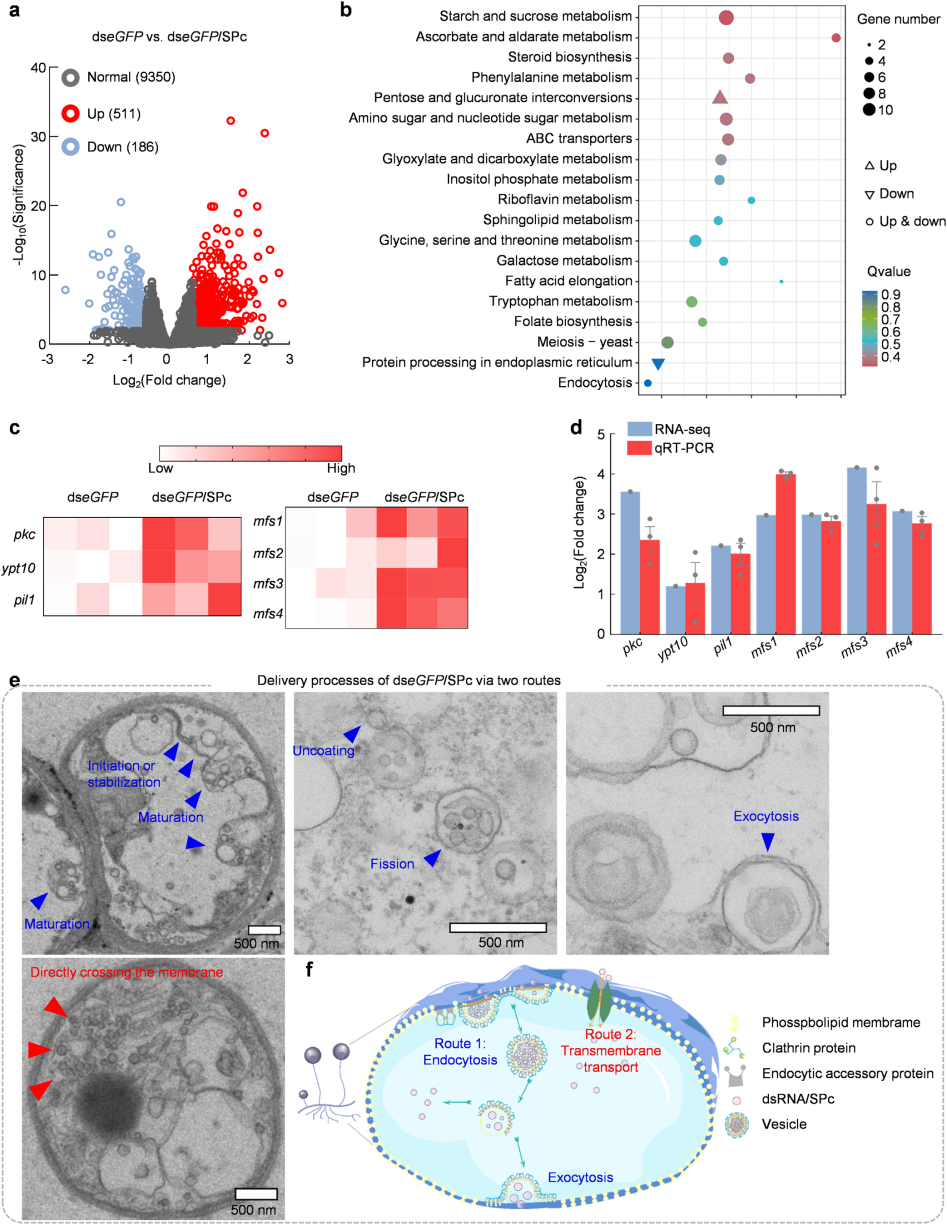
Enhanced delivery of SPc‐loaded dsRNA via activating endocytosis and transmembrane transport in *Botrytis cinerea* . (a–c) RNA‐seq analysis to illustrate the mechanism underling SPc‐mediated ds*eGFP* delivery. The mycelia were incubated with ds*eGFP* or ds*eGFP*/SPc complex to prepare RNA sequencing libraries (*n* = 3 replications). (a) Analysis of DEGs with volcano plot. Red and blue dots represent the up‐ and down‐regulated genes, respectively. DEG screening was based on |log_2_ (fold change) | ≥ 1.5. (b) KEGG enrichment of DEGs. (c) Heat maps of DEGs associated with endocytosis and transmembrane transport. Genes with high expression levels are shown in red, while those with low expression levels appear in white. (d) Validation of DEGs using the qRT‐PCR. Bar represents mean ± SEM. (e) Representative image for delivery processes of ds*eGFP*/SPc complexes via two routes. The route 1 and 2 are represented by blue and red triangles, respectively. (f) Schematic diagram for delivery processes of ds*eGFP*/SPc complexes via two routes.

For more detailed information, we further compared the DEGs in hyphae treated with ds*eGFP* and ds*eGFP*/SPc complex. A total of 697 DEGs were identified with 511 up‐regulated and 186 down‐regulated genes (Figure 3a). KEGG analysis revealed that the DEGs were enriched in various signal pathways associated with endocytosis, ABC transporters, meiosis, starch and sucrose metabolism, etc. (Figure 3b). More specifically, the incubation with ds*eGFP*/SPc complexes resulted in a significant up‐regulation of endocytosis‐related genes, including *pkc*, *ypt10* and *pil1* (Figure 3c). Additionally, the genes associated with transmembrane transport (*mfs1*, *mfs2*, *mfs3* and *mfs4*) were also activated, and these genes have MFS domain. The gene symbols based on their functional domains are shown in Table S2. Additionally, the qRT‐PCR results confirmed that the expression levels of tested seven DEGs aligned with the RNA‐seq data, supporting the reliability and reproducibility of RNA‐seq results (Figure 3d).

The DEGs were enriched in two primary routes for the translocation of exogenous substances across the cell membrane. According to previous studies, the mechanisms for absorbing exogenous dsRNA in insect and plant cells primarily involve endocytosis (Zhou, Jiang, et al. 2023; Zhou, Wang, et al. 2023; Yang et al. 2022; Ma et al. 2022). Three DEGs such as *pkc*, *ypt10* and *pil1* are related with the clathrin‐dependent or independent endocytosis (Figure S5a). The *pkc* comprises various isoforms and isoenzymes, widely implicated in multiple cellular functions, including regulatory roles in endocytosis (Holm et al. 1995). The *ypt10* acts as a small GTPase primarily involved in vesicle formation, microtubule‐ and actin‐dependent vesicle transport, and membrane fusion (Stenmark et al. 1995). The *pil1* is a crucial component of eisosomes, forming punctate clusters on the cytoplasmic surface of plasma membrane, commonly used as markers for endocytic sites (Swaminathan 2006; Walther et al. 2006). Therefore, these up‐regulated DEGs might facilitate the efficient delivery of SPc‐loaded dsRNA.

The DEGs were also enriched in another crucial route for the delivery of exogenous substances. Four DEGs such as *mfs1*, *mfs2*, *mfs3* and *mfs4* were associated with transmembrane transport (MFS transporter and ABC transporter) (Figure S5b). The *mfs2* and *mfs4* contained MFS domain, but they were not annotated in the KEGG enrichment. The MFS and ABC are the largest known secondary active transmembrane transport families, responsible for transporting a broad spectrum of substances, including ions, sugars, phosphates, drugs, nucleotides, etc. across the cell membranes (Yu et al. 2015; Lambert et al. 2022). However, there are very limited publications on transmembrane transport‐mediated dsRNA delivery in fungi. The MFS transporters facilitate the direct penetration of drug metabolites through the inner membrane in fungi (Park et al. 2019). Similarly, the ABC transporter can regulate the uptake of hydrophilic compounds in pathogenic bacteria (Rempel et al. 2020). Overall, the RNA‐seq results suggested that the application of SPc could activate two routes: endocytosis and transmembrane transport for enhanced uptake of dsRNA by mycelia.

To further verify the activation of above routes, the delivery process of SPc‐loaded dsRNA was visualized using the biological TEM. The typical endocytic process mainly includes five phases: initiation, stabilization, maturation, fission and uncoating (Mousavi et al. 2004). The initiation, stabilization and maturation phases mainly involve coat proteins aggregating to recognize cargo, gathering the actin to generate pulling force into the membrane, and finally breaking away from the plasma membrane to form a vesicle (Wu et al. 2023). During the fission and uncoating phases, the protein‐coated cave encloses the matured cargo vesicle, and coat proteins and actin are removed to release cargos (Varkouhi et al. 2011). All above phases could be observed with SPc‐loaded dsRNA in the current study (Figure 3e). During the initiation and stabilization phases, ds*eGFP*/SPc complexes were concentrated at the membrane surface, appearing either as single particles or clusters, while the membrane exhibited a concave shape. In the maturation phase, the inner membrane further invaginated to internalize ds*eGFP*/SPc complexes, and the formed vesicle gradually moved away from the plasma membrane. In the fission and uncoating phases, the vesicle containing ds*eGFP*/SPc complexes fully detached from the plasma membrane, and the ds*eGFP*/SPc complexes escaped from the vesicle. Furthermore, the exocytosis was also observed, and the vesicle moved to the plasma membrane and was transported out of the fungal cell. The typical transmembrane transport‐mediated delivery (MFS and ABC transporters) is also critical for the translocation of exogenous substances into the cytoplasm (Zhou et al. 2020). Our TEM images also showed that some ds*eGFP*/SPc complexes directly entered into the cytoplasm without the formation of vesicles, which might be regulated by MFS and ABC transporters. More representative TEM images were provided to verify above processes (Figure S6a), and two routes for the translocation of ds*eGFP*/SPc complexes were summarized in Figure 3f.

The SPc‐mediated endocytosis was quantified by counting vesicle number and measuring vesicle area. As shown in Figure S6b, more vesicles and larger vesicle area were present in the fungal cells incubated with ds*eGFP*/SPc complexes compared to naked ds*eGFP*. More specifically, the number of cargo‐containing vesicles increased from 4.43 to 16.57, and the relative vesicle area increased to 3.19, indicating stronger endocytosis with the aid of SPc. The particle size of ds*eGFP*/SPc complexes was 193.3 nm, and the number of particles with a similar size was recorded to quantify the transmembrane transport‐mediated delivery. The particle number increased from 20.40 to 38.20 in the mycelia incubated with ds*eGFP*/SPc complexes compared to naked ds*eGFP*. However, the naked ds*eGFP* was linear, and the particles in mycelia incubated with naked ds*eGFP* were unlikely to be ds*eGFP*. A previous study has confirmed that the particles with a size < 200 nm can efficiently penetrate the cell membrane to enter into the cytoplasm (Chen et al. 2016). Based on the above results, the SPc could accelerate the ds*eGFP* delivery in mycelia via two routes: endocytosis and transmembrane transport. Nevertheless, the key genes associated with the SPc‐mediated activation of the two routes should be further identified.

### 2.4 Crucial Genes in Two Routes for SPc‐Mediated dsRNA Delivery Enhancement

**FIGURE 4:**
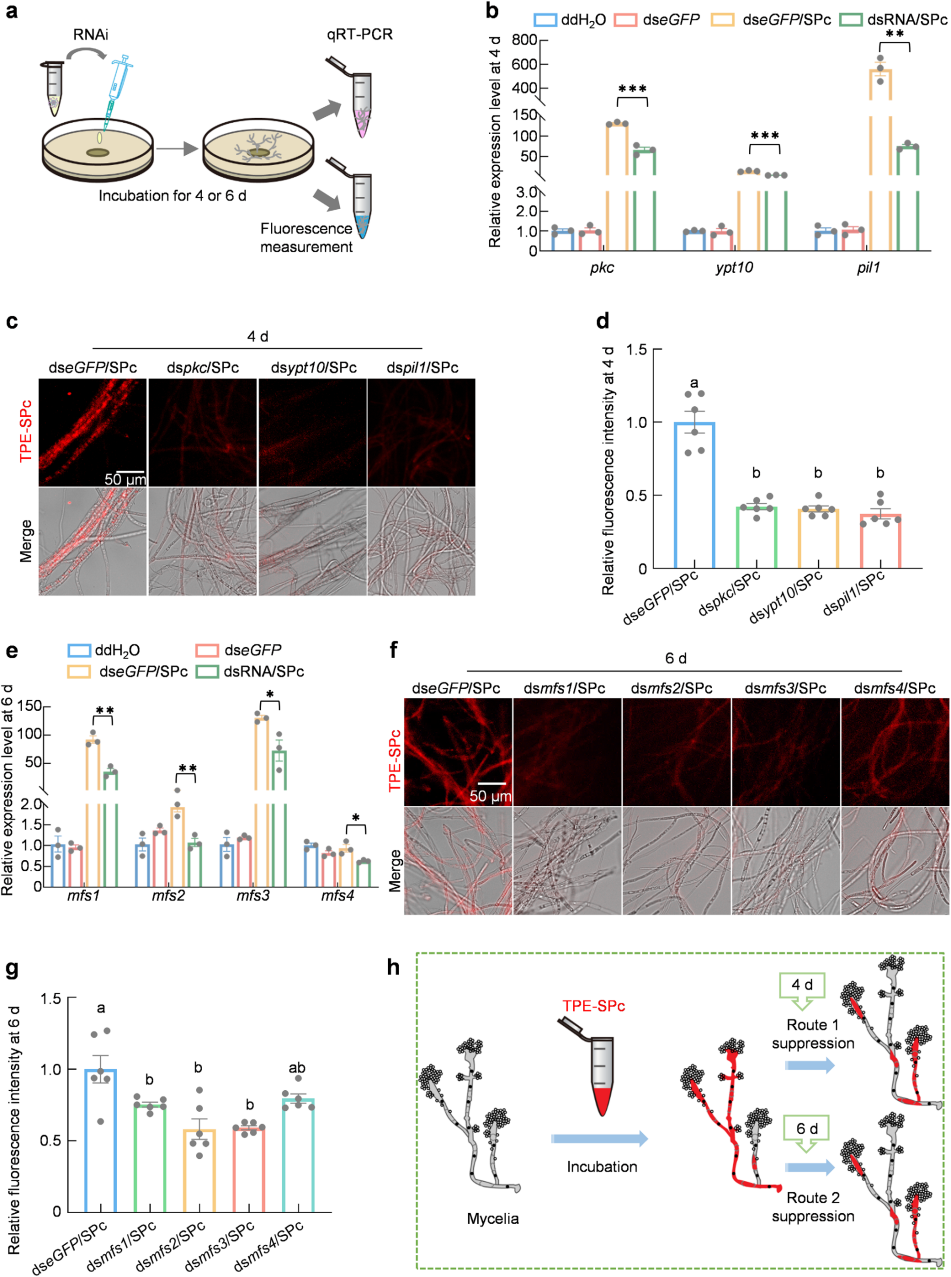
Crucial genes associated with SPc‐mediated dsRNA delivery enhancement in two routes. (a) Schematic diagram of experimental procedure. The mycelial discs were incubated with SPc‐loaded dsRNA and cultured for 4 or 6 days, and the RNAi efficiency of seven target genes and uptake efficiency of TPE‐SPc were examined. (b) Expression levels of *pkc*, *ypt10* and *pil1* in route 1 endocytosis after the incubation with the corresponding dsRNA (*n* = 3 replications). The asterisk indicates significant difference according to independent *t*‐test (two‐tailed, **p < 0.01, ***p < 0.001). *pkc* (*t* = 9.25, df = 4, *p* = 0.0008), *ypt10* (*t* = 9.97, df = 4, *p* = 0.0006) and *pil1* (*t* = 8.48, df = 4, *p* = 0.0011). (c) Representative fluorescence images of cellular uptake of TPE‐SPc by mycelia treated with dsRNA in route 1. (d) Relative fluorescence intensity of TPE‐SPc in mycelia after the incubation (*n* = 6 replications). Fluorescence intensity was quantified using ImageJ 1.8 software and normalized to control intensity values. Different letters above each bar indicate significant differences at *p* < 0.05 as determined by one‐way ANOVA with Tukey HSD test (*F*_3,20_ = 46.77, *p* < 0.0001). (e) Expression levels of *mfs1*, *mfs2*, *mfs3* and *mfs4* in route 2 transmembrane transport after the incubation with the corresponding dsRNA (*n* = 3 replications). The asterisk indicates significant difference according to independent *t*‐test (two‐tailed, *p < 0.05, **p < 0.01). *mfs1* (*t* = 6.64, df = 4, *p* = 0.0027), *mfs2* (*t* = 5.61, df = 4, *p* = 0.0050), *mfs3* (*t* = 3.04, df = 4, *p* = 0.0383) and *mfs4* (*t* = 3.41, df = 4, *p* = 0.027). (f) Representative fluorescence images of cellular uptake of TPE‐SPc by mycelia treated with dsRNA in route 2. (g) Relative fluorescence intensity of TPE‐SPc in mycelia after the incubation (*n* = 6 replications). Fluorescence intensity was quantified using ImageJ 1.8 software and normalized to control intensity values. Different letters above each bar indicate significant differences at *p* < 0.05 as determined by one‐way ANOVA with Tukey HSD test (*F*_4,25_ = 9.42, *p* < 0.0001). (h) Schematic diagram for uptake suppression after the treatment with SPc‐loaded dsRNA in route 1 and route 2. (b, d, e, g) Bar represents mean ± SEM.

The above genes in each route were individually down‐regulated via RNAi to identify potential mediating genes. The *B. cinerea* mycelia incubated with dsRNA/SPc complexes were harvested to examine the RNAi efficiency, and the TPE‐SPc was further added to the mycelia to examine the uptake efficiency (Figure 4a). For the endocytosis route, the down‐regulation of *pkc*, *ypt10* and *pil1* could be detected at 4 days after the incubation (Figure 4b). As shown in Figure 4c,d, the uptake of TPE‐SPc was inhibited in the mycelia after the down‐regulation of *pkc*, *ypt10* or *pil1*, and the relative fluorescence intensities decreased to 42%, 41% and 37%. A previous publication has revealed that the clathrin heavy chain gene (*CHC*) plays an important role in clathrin‐dependent endocytosis, and its suppression decreases the uptake of chitosan/dsRNA nanocomplexes by mites (Zhou, Jiang, et al. 2023; Zhou, Wang, et al. 2023). Similarly, the RNAi efficiency reduces after the RNAi of two core genes related to clathrin‐dependent endocytosis in aphids (Ye et al. 2021). The current study identified three crucial genes (*pkc*, *ypt10* and *pil1*) for SPc‐mediated endocytosis enhancement.

For transmembrane transport route, the expression levels of *mfs1*, *mfs2*, *mfs3* and *mfs4* could be down‐regulated at 6 days after the incubation, which was 2 days later than the endocytosis route (Figure 4e). The uptake efficiency of TPE‐SPc was lower in the mycelia after the down‐regulation of *mfs1*, *mfs2* or *mfs3*, and relative fluorescence intensities decreased to 75%, 58% and 59%, indicating the crucial function of three genes in transmembrane transport‐mediated dsRNA delivery (Figure 4f,g). The aforementioned results were summarised in Figure 4h. The inhibition of endocytosis route could be achieved in a relatively short period (4 days) to decrease the cellular uptake of dsRNA, and the suppression of transmembrane transport route could be achieved at 6 days for reduced uptake. Two routes may display complementary function for the cellular uptake of exogenous substances. Thus, the fungi may evolve some mechanisms to link two routes to avoid their long‐term co‐inhibition for survival.

### 2.5 Interaction Effect Between Two Routes for Cellular Uptake of SPc‐Loaded dsRNA

**FIGURE 5:**
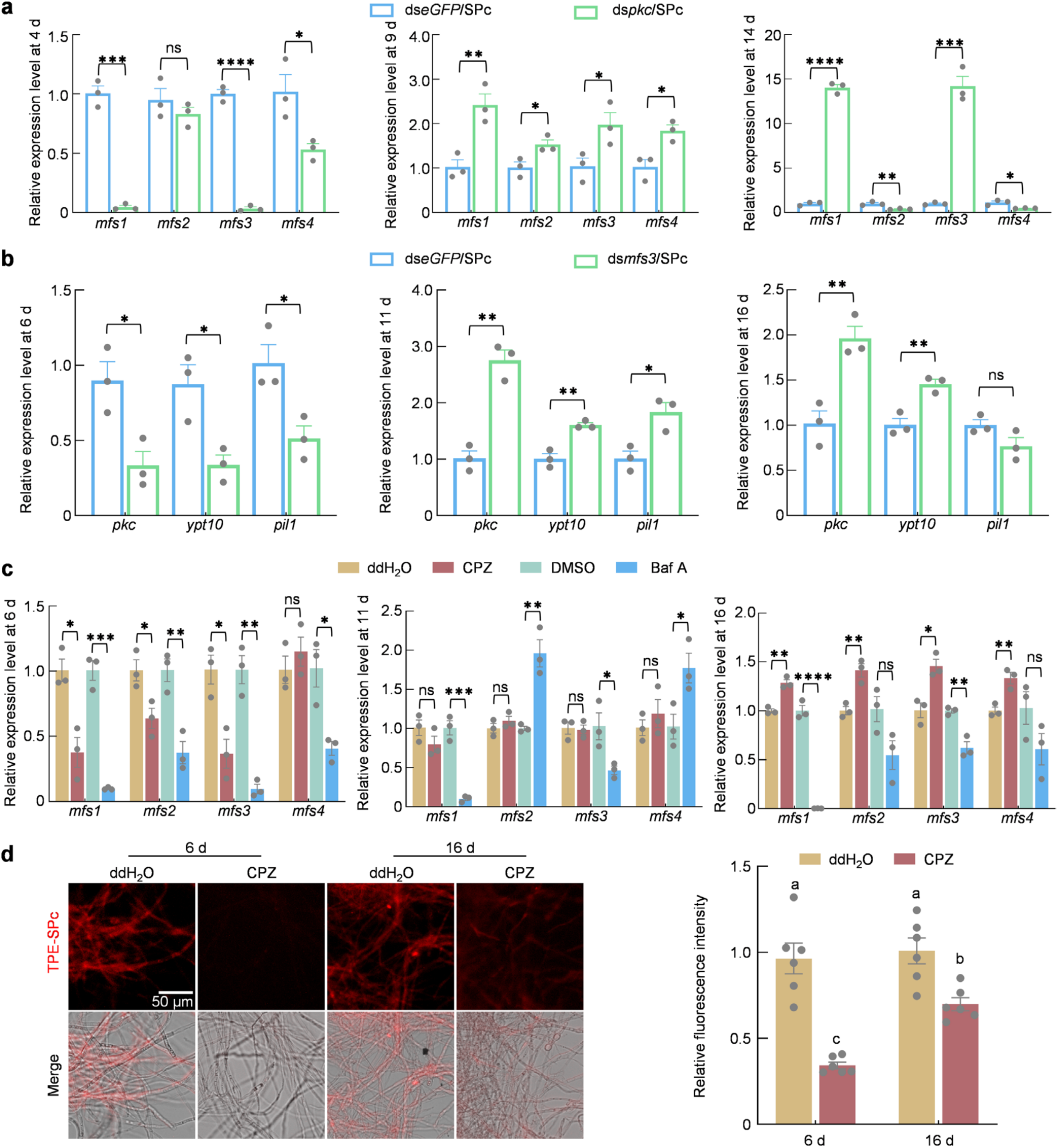
Interaction between two routes for cellular uptake of SPc‐loaded dsRNA. (a) Expression levels of four genes in route transmembrane transport at 4, 9 and 14 days after the RNAi of *pkc* (*n* = 3 replications). The asterisk indicates significant difference according to independent *t*‐test (two‐tailed; ns, no significance, *p < 0.05, **p < 0.01, ***p < 0.001, ****p < 0.0001). Four days: *mfs1* (*t* = 14.60, df = 4, *p* = 0.0001), *mfs2* (*t* = 1.07, df = 4, *p* = 0.3426), *mfs3* (*t* = 24.61, df = 4, *p* < 0.0001) and *mfs4* (*t* = 3.19, df = 4, *p* = 0.0329); 9 d: *Mfs1* (*t* = 4.66, df = 4, *p* = 0.0096), *mfs2* (*t* = 3.12, df = 4, *p* = 0.0354), *mfs3* (*t* = 2.80, df = 4, *p* = 0.0488) and *mfs4* (*t* = 3.84, df = 4, *p* = 0.0183); 14 days: *mfs1* (*t* = 34.71, df = 4, *p* < 0.0001), *mfs2* (*t* = 4.73, df = 4, *p* = 0.0091), *mfs3* (*t* = 11.77, df = 4, *p* = 0.0003) and *mfs4* (*t* = 3.93, df = 4, *p* = 0.0170). (b) Expression levels of three genes in endocytosis route at 6, 11 and 16 d after the RNAi of *mfs3* (*n* = 3 replications). The asterisk indicates significant difference according to independent *t*‐test (two‐tailed; ns, no significance, *p < 0.05, **p < 0.01). Six days: *pkc* (*t* = 3.62, df = 4, *p* = 0.0223), *ypt10* (*t* = 3.74, df = 4, *p* = 0.0201) and *pil1* (*t* = 3.34, df = 4, *p* = 0.0287); 11 days: *pkc* (*t* = 7.69, df = 4, *p* = 0.0015), *ypt10* (*t* = 6.13, df = 4, *p* = 0.0036) and *pil1* (*t* = 3.79, df = 4, *p* = 0.0191); 16 days: *pkc* (*t* = 4.92, df = 4, *p* = 0.0079), *ypt10* (*t* = 5.15, df = 4, *p* = 0.0067) and *pil1* (*t* = 2.12, df = 4, *p* = 0.1009). (c) Expression levels of four genes in route transmembrane transport at 6, 11 and 16 days after the incubation with CPZ or Baf A (*n* = 3 replications). The asterisk indicates significant difference according to independent *t*‐test (two‐tailed; ns, no significance, *p < 0.05, **p < 0.01, ***p < 0.001, ****p < 0.0001). 6 days: CPZ versus ddH_2_O, *mfs1* (*t* = 4.40, df = 4, *p* = 0.0116), *mfs2* (*t* = 3.26, df = 4, *p* = 0.0309), *mfs3* (*t* = 4.11, df = 4, *p* = 0.0146) and *mfs4* (*t* = 0.90, df = 4, *p* = 0.4154); Baf A versus DMSO, *mfs1* (*t* = 12.02, df = 4, *p* = 0.0003), *mfs2* (*t* = 5.19, df = 4, *p* = 0.0066), *mfs3* (*t* = 8.02, df = 4, *p* = 0.0013) and *mfs4* (*t* = 4.00, df = 4, *p* = 0.0160); 11 days: CPZ versus ddH_2_O, *mfs1* (*t* = 1.48, df = 4, *p* = 0.2123), *mfs2* (*t* = 1.12, df = 4, *p* = 0.3252), *mfs3* (*t* = 0.22, df = 4, *p* = 0.8312) and *mfs4* (*t* = 0.88, df = 4, *p* = 0.4276); Baf A versus DMSO, *mfs1* (*t* = 9.78, df = 4, *p* = 0.0006), *mfs2* (*t* = 5.47, df = 4, *p* = 0.0054), *mfs3* (*t* = 3.12, df = 4, *p* = 0.0355) and *mfs4* (*t* = 3.02, df = 4, *p* = 0.0388); 16 days: CPZ versus ddH_2_O, *mfs1* (*t* = 7.20, df = 4, *p* = 0.0020), *mfs2* (*t* = 5.13, df = 4, *p* = 0.0068), *mfs3* (*t* = 4.41, df = 4, *p* = 0.0116) and *mfs4* (*t* = 4.77, df = 4, *p* = 0.0088); Baf A versus DMSO, *mfs1* (*t* = 18.93, df = 4, *p* < 0.0001), *mfs2* (*t* = 2.40, df = 4, *p* = 0.0742), *mfs3* (*t* = 5.72, df = 4, *p* = 0.0046) and *mfs4* (*t* = 1.83, df = 4, *p* = 0.1408). (d) Fluorescence intensities of TPE‐SPc in mycelia incubated with CPZ for 6 and 16 days (*n* = 6 replications). Fluorescence intensity was quantified using ImageJ 1.8 software and normalized to control intensity values. Different letters above each bar indicate significant differences at *p* < 0.05 as determined by one‐way ANOVA with Tukey HSD test (*F*_3,20_ = 24.08, *p* < 0.0001). (a–d) Bar represents mean ± SEM.

Above results revealed that the short‐term suppression (RNAi) of either route was sufficient to inhibit the cellular internalization of dsRNA/SPc complexes, indicating the presentation of an interaction effect between two routes. Whereas the long‐term co‐inhibition of two routes may be able to severely hinder the cellular uptake of nutrients, which adversely influences the normal growth of organisms. Thus, the interaction effect between two routes may be very complicated, which has not been explored and demonstrated. In the current study, the shot‐ and long‐term suppression of one route was achieved via RNAi and chemical inhibitors, and their effects on another route were extensively analysed. Short‐term RNAi (4 days) of *pkc* could down‐regulate the expression levels of most tested genes in the transmembrane transport route, whereas its long‐term RNAi (9 and 14 days) activated the transmembrane transport route (Figure 5a). Similarly, the short‐term down‐regulation of *mfs3* in the transmembrane transport route could inhibit the expression of all tested genes in the endocytosis route, and the expression levels of genes in the endocytosis route were up‐regulated after persistent RNAi (Figure 5b). Two routes are regarded as independent pathways for the cellular uptake of dsRNA, but our results demonstrated their interaction at least at the transcriptional level. Chlorpromazine (CPZ) and bafilomycin‐A (Baf A) that can inhibit clathrin‐dependent endocytosis (Saleh et al. 2006; Xiao et al. 2015), were applied to suppress the endocytosis to examine the interaction effects between two routes. As shown in Figure 5c, the short‐term exposure to CPZ or Baf A could suppress the expression of most genes in the transmembrane transport route, whereas its long‐term incubation activated the transmembrane transport route. Additionally, short‐term (6 days) incubation with CPZ could decrease the cellular uptake of TPE‐SPc with the reduction in fluorescence intensity to 34%, but the fluorescence intensity increased to 70% after long‐term (16 days) exposure to CPZ (Figure 5d). The expression of *mfs1*, *mfs2*, *mfs3* and *mfs4* not associated with the endocytosis was also significantly altered, indicating that the two routes interacted with each other. Additionally, the specific chemical inhibitors need further verification (Daniel et al. 2015; Wang et al. 1993).

**FIGURE 6:**
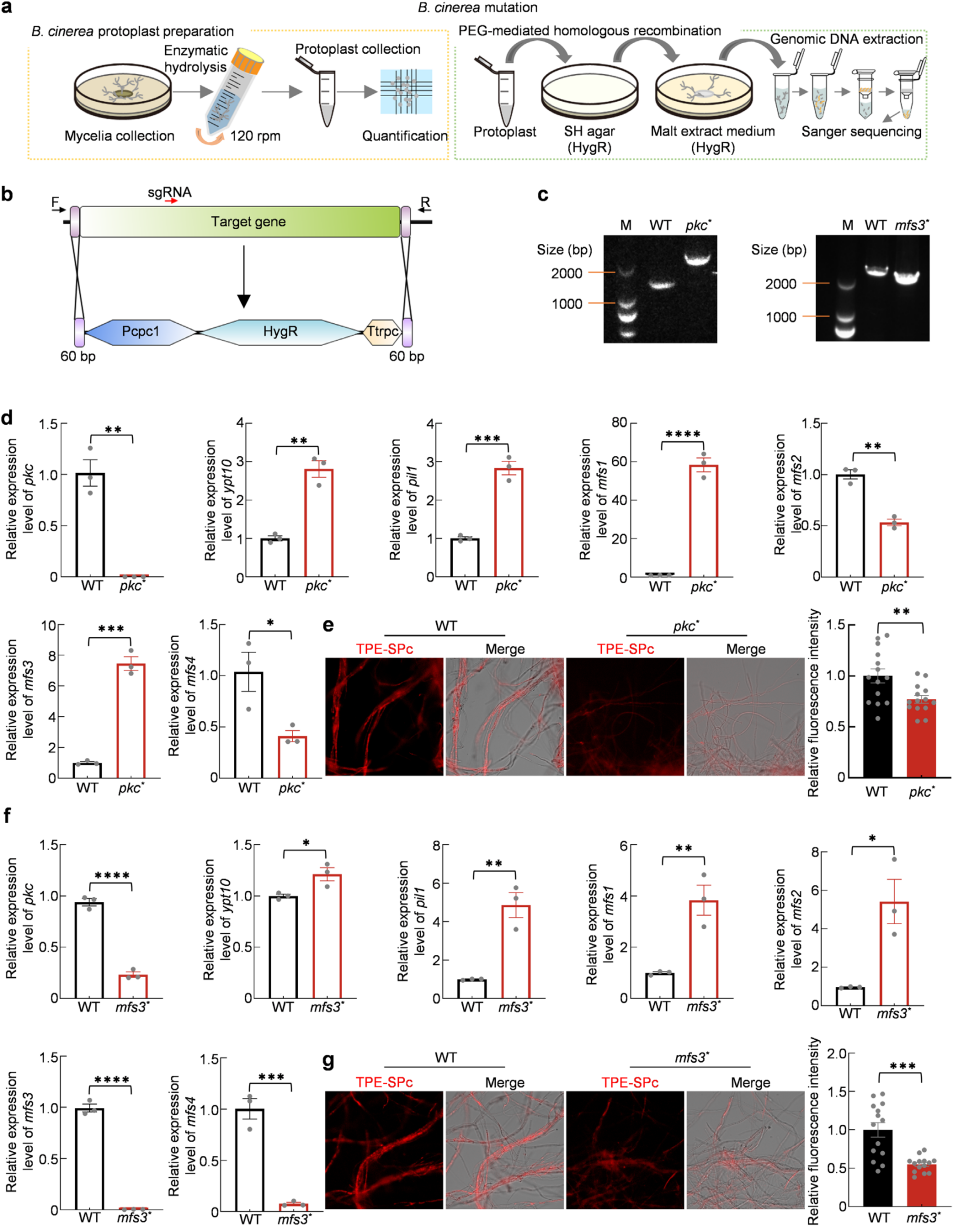
Cellular uptake inhibition after the knockout of *pkc* or *mfs3* via affecting the gene expression in two routes. (a) Schematic diagram for the transformation process of *Botrytis cinerea* . (b) Construction of *B. cinerea* mutant strains. The direction of sgRNA is indicated by red arrow. Homologous arm regions were designed with a length of 60 bp. Primers for validating transformants are labelled as F or R. The target gene was replaced with the Pcpc1‐HygR‐Ttrpc. (c) Validation of positive transformants with *pkc* and *mfs3* mutations (pkc* and mfs3* strains). (d) Expression levels of seven genes in two routes endocytosis and transmembrane transport in the pkc* strain (*n* = 3 replications). The asterisk indicates significant difference according to independent *t*‐test (two‐tailed: *p < 0.05, **p < 0.01, ***p < 0.001, ****p < 0.0001). *pkc* (*t* = 7.90, df = 4, *p* = 0.0014), *ypt10* (*t* = 8.05, df = 4, *p* = 0.0013), *pil1* (*t* = 10.07, df = 4, *p* = 0.0005). *mfs1* (*t* = 15.99, df = 4, *p* < 0.0001), *mfs2* (*t* = 8.42, df = 4, *p* = 0.0011), *mfs3* (*t* = 14.34, df = 4, *p* = 0.0001) and *mfs4* (*t* = 3.17, df = 4, *p* = 0.0339). (e) Fluorescence intensities of TPE‐SPc in WT and pkc* strains. Each treatment contained 14 independent images. Fluorescence intensity was quantified using ImageJ 1.8 software and normalized to control intensity values. The asterisk indicates significant difference according to independent *t*‐test (two‐tailed: *t* = 2.90, df = 26, *p* = 0.0075). (f) Expression levels of seven genes in two routes in the mfs3* strain (*n* = 3 replications). The asterisk indicates significant difference according to independent *t*‐test (two‐tailed: *p < 0.05, **p < 0.01, ***p < 0.001, ****p < 0.0001). *pkc* (*t* = 16.07, df = 4, *p* < 0.0001), *ypt10* (*t* = 3.20, df = 4, *p* = 0.033), *pil1* (*t* = 5.94, df = 4, *p* = 0.004). *mfs1* (*t* = 4.85, df = 4, *p* = 0.0084), *mfs2* (*t* = 3.87, df = 4, *p* = 0.018), *mfs3* (*t* = 25.37, df = 4, *p* < 0.0001) and *mfs4* (*t* = 9.15, df = 4, *p* = 0.0008). (g) Fluorescence intensities of TPE‐SPc in WT and mfs3* strains. Each treatment contained fourteen independent images. Fluorescence intensity was quantified using ImageJ 1.8 software and normalized to control intensity values. The asterisk indicates significant difference according to independent *t*‐test (two‐tailed: *t* = 4.58, df = 26, *p* = 0.0001). (d–g) Bar represents mean *±* SEM.

The crucial genes *pkc* and *mfs3* in two routes were selected as targets to construct mutant strains by gene edition technique to verify the long‐term interaction between two routes, which mainly involved protoplast preparation and PEG‐mediated homologous recombination (Figure 6a). The single‐guide RNA (sgRNA) fragments were verified through in vitro cleavage assays with CRISPR‐Cas9. Compared to the band of *pkc* or *mfs3*, the bands were effectively degraded after the incubation of sgRNA with Cas9 (Figure S7a). The homologous sequence Pcpc1‐Hygr‐Ttrpc was employed to replace the *pkc* and *mfs3* (Table S4; Figure 6b). Genomic DNA was extracted to validate the positive colony (Figure 6c). In the pkc* strain (*pkc* mutant), the important genes in the endocytosis route (*ypt10* and *pil1*) and transmembrane transport route (*mfs1* and *mfs3*) were significantly up‐regulated, and the uptake efficiency of TPE‐SPc was much lower than that in wide‐type (WT) strain (Figure 6d,e). In the mf3* strain, the crucial genes *ypt10*, *pil1*, *mfs1* and *mfs2* were also significantly up‐regulated, and the cellular uptake of TPE‐SPc decreased (Figure 6f,g). The expression patterns of seven genes in two routes were similar in the mutants from those after the long‐term RNAi. The knockout of *pkc* or *mfs3* had a more long‐term adverse impact on fungi, and the growth status of the mutant strain was not as good as the wild strain (Figure S7b). The shutdown of the endocytosis route or transmembrane transport route might promote the fungi to activate alternative pathways to maintain normal cellular uptake. Thus, the interaction between two routes is complicated, and upstream and downstream relationships of genes, as well as the regulatory network between pathways, need further exploration. Based on the current results, the SPc could improve dsRNA delivery via two interactional routes, which could be applied as a nanocarrier to develop high‐efficiency RNA fungicides.

### 2.6 Excellent Protective Effects of SPc‐Based RNA Fungicides Toward Grey Mould Disease

**FIGURE 7:**
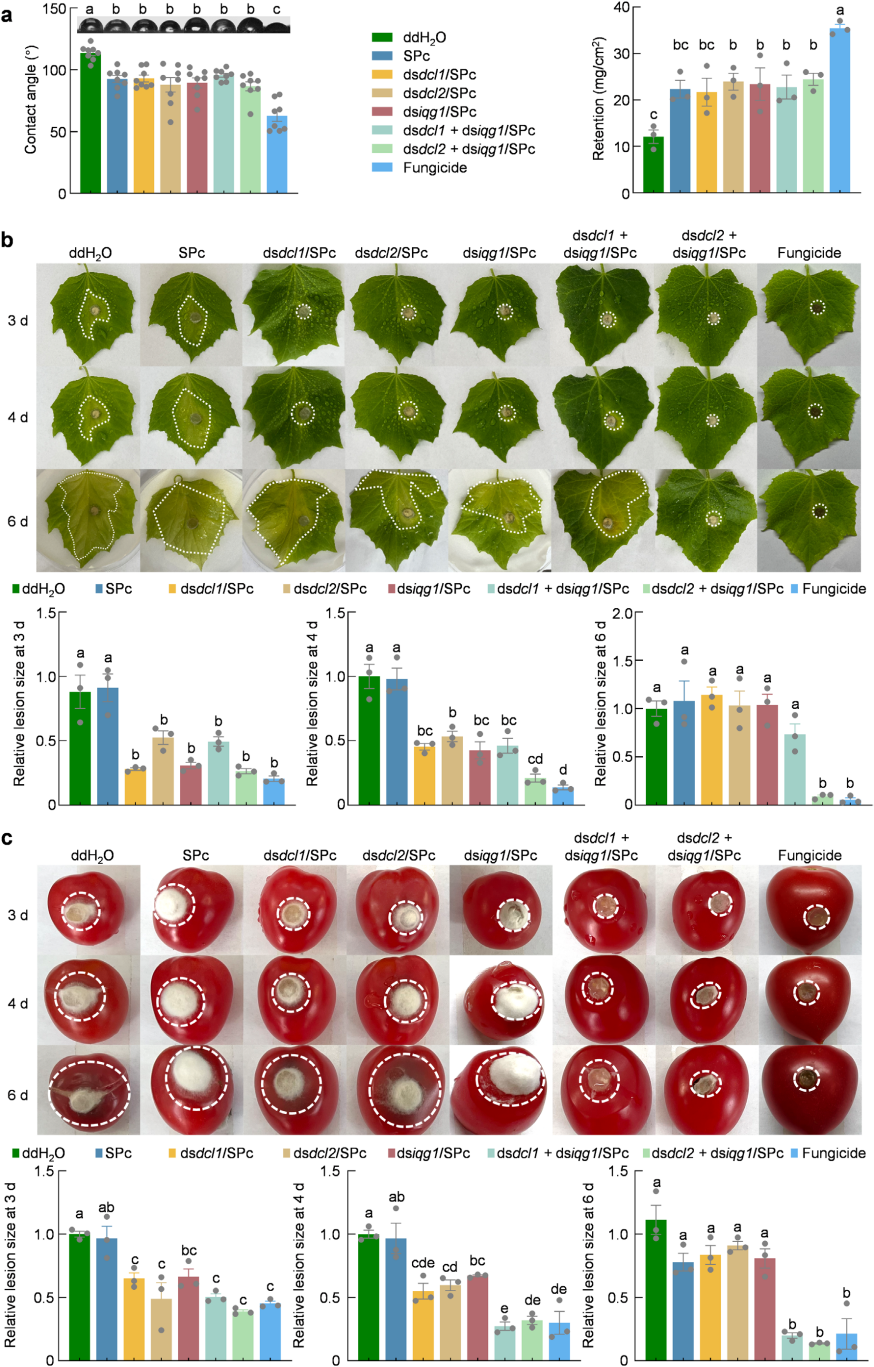
Excellent adhesion performance and protective effect of SPc‐based RNA fungicides toward grey mould disease. (a) Contact angles (*n* = 8 replications) and retentions (*n* = 3 replications) of various solutions on cucumber leaves. Different letters above each bar indicate significant differences at *p* < 0.05 as determined by one‐way ANOVA with Tukey HSD test (Contact angle: *F*_7,56_ = 15.03, *p* < 0.0001; Retention: *F*_7,16_ = 8.12, *p* = 0.0003). (b) Protective effects of SPc‐loaded dsRNA on cucumber leaves. Incisions were made on the leaves, and various formulations were sprayed on the leaves, which were then inoculated with *Botrytis cinerea* mycelium. Images were captured at 3, 4 and 6 days after the inoculation, and the lesion sizes were measured using ImageJ 1.8 software (*n* = 3 replications). Different letters above each bar indicate significant differences at *p* < 0.05 as determined by one‐way ANOVA with Tukey HSD test (3 days: *F*_7,16_ = 18.12, *p* < 0.0001; 4 days: *F*_7,16_ = 29.26, *p* < 0.0001; 6 days: *F*_7,16_ = 15.60, *p* < 0.0001). (c) Protective effects of SPc‐loaded dsRNA on tomato fruits. Holes were made in the centre of fruits, and various formulations were sprayed on the fruits, which were then inoculated with *B. cinerea* mycelium. Images were captured at 3, 4 and 6 days after the inoculation, and the lesion sizes were measured using ImageJ 1.8 software (*n* = 3 replications). Different letters above each bar indicate significant differences at *p* < 0.05 as determined by one‐way ANOVA with Tukey HSD test (3 days: *F*_7,16_ = 13.26, *p* < 0.0001; 4 days: *F*_7,16_ = 20.90, *p* < 0.0001; 6 days: *F*_7,16_ = 25.29, *p* < 0.0001). (a–c) Bar represents mean ± SEM.

Three crucial genes (*dcl1*, *dcl2* and *iqg1*) were employed as RNAi targets for developing RNA fungicides toward destructive grey mould disease (Wang and Jin 2017; Xu et al. 2024). Due to the enhanced stability and delivery of SPc‐loaded dsRNA, the dsRNA/SPc complex could significantly suppress the expression of *dcl1*, *dcl2* and *iqg1* compared to naked dsRNA (Figure S8). To evaluate the protective effects of RNA fungicides, a commercial fungicide (1.5% osthole‐matrine) was introduced as a positive control. The wettability and retention of leaves are crucial factors for effective control of grey mould diseases (Nerva et al. 2020). Compared to ddH_2_O, the contact angles of all tested formulations significantly decreased on cucumber leaves, and their retention increased, indicating the excellent adhesion performance of RNA fungicides (Figure 7a).

A series of RNA fungicides were sprayed onto cucumber leaves and tomato fruits to evaluate their protective effects with ddH_2_O and SPc as negative controls and commercial fungicide (higher concentration than recommendation) as positive control. Among tested RNA fungicides, the ds*dcl2* + *dsiqg1*/SPc complexes displayed the best protective effects on cucumber leaves, which was comparable to commercial fungicide. The relative lesion sizes on leaves treated with ds*dcl2* + *dsiqg1*/SPc complexes reduced to 29%, 21% and 9.2% at 3, 4 and 6 days compared to those with ddH_2_O (Figure 7b). Similarly, the protective effects of ds*dcl2* + *dsiqg1*/SPc complexes and commercial fungicide were excellent on tomato fruits, and the relative lesion sizes on fruits treated with ds*dcl2* + *dsiqg1*/SPc complexes reduced to 39%, 32% and 13% at 3, 4 and 6 days compared to those with ddH_2_O (Figure 7c). As expected, all corresponding RNA fungicides without SPc showed no protective effects on cucumber leaves and tomato fruits, confirming the important function of SPc in developing RNA fungicides (Figure S9). The SPc was compared with established nano‐delivery systems, such as BioClay and artificial vesicles, to evaluate its potential as an alternative delivery carrier from a broader perspective. By contrast, while the pioneering BioClay platform extends protection against *B. cinerea* by delivering exogenous dsRNA into plants, its residual LDH accumulates within cells and resists washing (Niño‐Sánchez et al. 2022; Mitter et al. 2017). In comparison, the hyphal cells could excrete SPc in the current study, providing a safer alternative in plants. Additionally, artificial vesicle/dsRNA complexes are typically larger in size (259 and 393 nm) and exhibit a higher binding ratio with dsRNA (4:1) (Qiao et al. 2023). In contrast, dsRNA/SPc complexes showed a significantly smaller average size (193 nm) with a superior binding mass ratio of 1:1. These characteristics indicated that the SPc not only possessed superior assembly efficiency but also that its smaller size could facilitate the better translocation across delivery barriers. In summary, our work presented a groundbreaking innovation: the SPc could not only protect dsRNA from degradation to increase its stability, but also activate two interactional routes endocytosis and transmembrane transport for enhanced dsRNA uptake. Above advantages provided an excellent nanoplatform for designing/developing novel high‐efficiency RNA fungicides toward destructive plant diseases, which promoted the practice and development of RNAi‐based pest management strategy.

## 3 Experimental Procedures

### 3.1 Fungal Strain and Culture Conditions

*Botrytis cinerea* was purchased from BeNa culture collection Co. (China), and cultured on potato dextrose agar (PDA) medium (Aobox Biotechnology Co., China) at room temperature.

### 3.2 Synthesis of dsRNA In Vitro

*Botrytis cinerea* mycelia were harvested from the PDA medium overlaid with cellophane (Solarbio Co., China) for RNA extraction. Total RNA was extracted using the RNA isolator Total RNA extraction reagent (Vazyme Co., China). Subsequently, the RNA was reverse‐transcribed into cDNA using the Prime Script RT reagent Kit with gDNA Eraser reagent (Takara Co., Japan). The resulting cDNA served as a template for amplifying the target genes, and the genes were cloned into pMD19T‐Vector (Takara Co.) and transformed into *E. coli* DH5α (Tsingke Co., China). The plasmid was extracted, verified by Sanger sequencing and used to synthesize the dsRNA following the T7 RNAi Transcription Kit instructions (Vazyme Co.). Fluorescent ds*eGFP* was synthesized using the synthesized T7‐DNA as a template, following the guidelines provided by Roche Fluorescence RNA Labeling Mix (Roche Co., USA), which could be detected at the wavelength of 488–520 nm. The synthetic protocol included the following components: 500 ng of T7‐*eGFP*, 2 μL of fluorescein RNA Labeling Mix, 2 μL of 10× transcription buffer, 2 μL of RNA polymerase (T7) and RNase‐free ddH_2_O to reach a total volume of 20 μL. The reaction mixture was thoroughly mixed and incubated at 37°C for 2 h, followed by a 10 min incubation at 70°C. Finally, 2 μL of 0.2 M EDTA terminates response was added. All primers (Table S3) were synthesized by the Tsingke Co.

### 3.3 Self‐Assembly Mechanism Assay of dsRNA/SPc Complex

The SPc was synthesized through two reaction steps according to previous methods (Li et al. 2019). The complexation of dsRNA with SPc was detected via two methods: gel electrophoresis and ITC. The ds*eGFP* was used to determine the optimal mass ratio for the complexation of dsRNA with SPc. The ds*eGFP* and SPc were dissolved in ddH_2_O and incubated at the mass ratios of 1:0, 2:1, 3:2, 1:1, 1:2 and 2:3 at room temperature for 15 min. The mixture was then analysed using the 2% agarose gel. The dsRNA was incubated with SPc at the mass ratio of 1:1 for the following experiment. Furthermore, zeta potential values of ds*eGFP*/SPc complex (ds*eGFP* concentration: 100 ng/μL), naked ds*eGFP* and SPc were measured using the Brookhaven NanoBrook Omni (USA) at 25°C.

The ITC provides an accurate determination of thermodynamic parameters governing the intermolecular interactions, thereby indicating the predominant interaction forces between two molecules. Specifically, 1 mL ds*eGFP* aqueous solution (0.0025 mM) was reacted with 250 μL SPc aqueous solution (0.000345 mM) in the ITC instrument (TA Instruments Waters, USA) at 25°C. The procedure of stabilization period was set to 300 s and 25 titration cycles. Then, the collected calorimetric data were integrated and analysed using the NanoAnalyze software (USA) to determine the interaction heat. The value of Δ*G* was calculated using the following equation:

(1) ΔG=ΔH–TΔS

### 3.4 Characterization Analysis of ds*eGFP*/SPc Complex

The morphologies of ds*eGFP*/SPc complex, ds*eGFP* and SPc were characterized using the TEM (JEOL‐1200, Japan) and AFM (Bruker Dimension ICON, Germany). For TEM, 10 μL sample was deposited onto a copper grid and dried completely. The grid was then negatively stained with uranyl acetate to enhance the electron scattering. For AFM, the above samples were individually diluted to 50 ng/μL with RNase‐free water and directly dropped onto a mica sheet for preparation. The surface morphology and roughness of the samples were then imaged. The average sizes of the above samples were measured using the Brookhaven NanoBrook Omni.

### 3.5 Stability Assay of dsRNA/SPc Complex Treated With RNase A

A previous method was modified to examine the protective effects of SPc on dsRNA (Wang, Li, Ying, et al. 2023; Wang, Li, Zhang, et al. 2023; Wang, Yan, Lan, et al. 2023). The 1 μg short/long dsRNA (ds*eGFP* 420 bp or ds*tublin* 1047 bp) and 1 μg SPc were incubated for 15 min to prepare the dsRNA/SPc complexes. Subsequently, 1 μL (100 ng/μL) of RNase A solution (Solarbio Co., China) was added, and the mixture was incubated at 37°C for 20 min. Finally, sodium dodecyl sulphate (SDS, Merck Co., China) at the final concentration of 0.5% was added, and the resulting solution was separated using 2% agarose gel. The relative fluorescence intensity of dsRNA was quantified using the ImageJ 1.8 software (USA). The naked dsRNA was employed as control, and each treatment included three independent samples.

### 3.6 Zeta Potential Measurement of *B. cinerea* Mycelia

To determine the surface charge of mycelia, 7‐day‐old mycelia were suspended in ddH_2_O, and its zeta potential value was measured using the Brookhaven NanoBrook Omni similarly as above.

### 3.7 Retention Assay of SPc‐Loaded ds*eGFP* on *B. cinerea* Mycelia

To evaluate the retention of ds*eGFP*/SPc complex, uniform mycelial discs (0.44 cm^2^) were weighed (*M*_1_) using a balance and immersed in the solutions of ds*eGFP*/SPc complex (ds*eGFP* concentration: 100 ng/μL), ds*eGFP* and SPc for 15 s, respectively. After immersion, the discs were removed with tweezers and weighed (*M*_2_) when excess liquid stopped dripping. The retention (mg/cm^2^) was calculated using the following formula:

(2) $$\text{Retention}\;\left(\mathrm{mg}/{\mathrm{cm}}^{2}\right)=\left(M_{2}-M_{1}\right)÷\text{disc area}$$

### 3.8 Uptake Efficiency Assay of SPc‐Loaded ds*eGFP* by *B. cinerea* Mycelia

The fluorescent groups were added into SPc to prepare the TPE‐SPc that could be detected at 365 nm (Zhou, Jiang, et al. 2023; Zhou, Wang, et al. 2023). The fluorescent ds*eGFP* and TPE‐SPc were incubated at the mass ratio of 1:1, and the mycelia were cultured in the solutions of ds*eGFP*/TPE‐SPc complex and ds*eGFP* at the final ds*eGFP* concentration of 20 ng/μL.

The absorption of ds*eGFP*/TPE‐SPc complex and ds*eGFP* by the mycelia was observed at 0.5, 6, 9 and 24 h after the culture using the inverted fluorescence microscope (EVOS FL, Advanced Microscopy Group, USA). Fluorescence intensities of ds*eGFP* and TPE‐SPc (red is a false colour) in the mycelia were quantified using the ImageJ 1.8 software. Additionally, the ds*eGFP* content in the mycelia was quantified using the qRT‐PCR (QuantStudio 1 Plus System, USA) according to a previous method (Ma et al. 2023). The qRT‐PCR conditions were as follows: an initial denaturation at 95°C for 3 min, succeeded by 40 cycles of denaturation at 95°C for 10 s, annealing at 56°C for 30 s and extension at 72°C for 30 s. The standard curve was plotted with cycle threshold (Ct) as ordinate and Log_10_(ds*eGFP* pg) as abscissa. The mycelia were incubated with the ds*eGFP*/SPc (without fluorescent signals) complex and ds*eGFP* solution for 6 h at the final ds*eGFP* concentration of 20 ng/μL. Subsequently, the mycelia were washed three times with ddH_2_O for RNA extraction, and the Ct values were obtained using the qRT‐PCR. The absolute content of ds*eGFP* in mycelia was then calculated using the standard curve.

### 3.9 Analysis for the Potential Mechanism Underling Enhanced Uptake of SPc‐Loaded ds*eGFP*

*Botrytis cinerea* mycelia were treated with ddH_2_O, SPc, ds*eGFP* or ds*eGFP*/SPc complex (the final ds*eGFP* concentration of 20 ng/μL) for 30 min. Total RNA was extracted according to the method described above and used to create an mRNA sequencing library. The Illumina NovaSeq6000 sequencing platform (Illumina, USA) was employed for PE150 mode sequencing. After obtaining the sequencing data, RNA‐seq data analysis was performed using the bioinformatics analysis pipeline provided by BMK Cloud (www.biocloud.net). The |log_2_ (fold change)| ≥ 1.5 between transcripts was used as the screening criterion for DEGs. The DEGs of ds*eGFP* versus ds*eGFP*/SPc complex were further validated using the qRT‐PCR to confirm the RNA‐seq results. The relative expression levels of DEGs were quantified using the 2^−ΔΔCt^ method, and the *actin* (XM_024697950.1) was used for normalization. The functional enrichment analysis was annotated using the KEGG databases.

The delivery processes of SPc‐loaded ds*eGFP* in *B. cinerea* mycelia were observed using the biological TEM (Hitachi HT7800, Japan). A 1 × 1 cm piece of mycelia was harvested and incubated with ds*eGFP*/SPc complex (ds*eGFP* concentration: 20 ng/μL) or naked ds*eGFP* for 30 min. Subsequently, the mycelia were fixed in 2.5% glutaraldehyde (Solarbio, China) at 4°C for 12 h. The mycelia were then rinsed with 0.1 M pH 7.0 phosphate buffer, and dehydration treatment was performed using ethanol solutions for 15 min. The samples were then treated with the embedding agent at 70°C overnight to obtain the embedded samples. An ultrathin microtome was used to slice the samples into 70–90 nm slices, which were stained with lead citrate solution and 50% ethanol‐saturated solution of uranyl acetate for 5–10 min. After dried, slices were observed using the TEM (HITACHI H‐7650).

### 3.10 Identification of Crucial Genes in Two Routes for the Enhanced Delivery of SPc‐Loaded dsRNA

The RNA‐seq results revealed that two routes (endocytosis and transmembrane transport) were activated in the mycelia treated with SPc‐loaded dsRNA, and the dsRNAs for DEGs such as *pkc*, *ypt10*, *pil1*, *mfs1*, *mfs2*, *mfs3* and *mfs4* in two routes were synthesized for RNAi according to a previous method (Bhagta et al. 2023). The dsRNA was incubated with SPc to prepare the dsRNA/SPc complexes, which were incubated with mycelial discs at the final dsRNA concentration of 100 ng/μL. The ds*eGFP* was employed as a control. At 4 or 6 days after the incubation, the total RNA was extracted to prepare the cDNA for qRT‐PCR to examine the RNAi efficiency in mycelia. Meanwhile, the mycelia were harvested at 4 or 6 days after the incubation, and then incubated with 50 μL TPE‐SPc solution (20 ng/μL) for 30 min. The uptake efficiency of TPE‐SPc by mycelia was examined using the inverted fluorescence microscope (EVOS FL).

### 3.11 Relationship Analysis of Two Routes for Enhanced Delivery of SPc‐Loaded dsRNA

To analyse the potential relationship of two routes, short‐ and long‐term RNAi methods were employed to examine the expression of genes in two routes. More specifically, the ds*pkc*/SPc or ds*mfs3*/SPc complex (dsRNA concentration: 100 ng/μL) was employed to incubate with the mycelial discs every 24 h. The above cDNA sample for *pkc* RNAi was employed to examine the expression of *mfs1*, *mfs2*, *mfs3* and *mfs4* at 4, 9 and 14 days using qRT‐PCR, and that for *mfs3* RNAi was employed to examine the expression of *pkc*, *ypt10* and *pil1* at 6, 11 and 16 days. Moreover, chemical inhibitors (CPZ and Baf A) of clathrin‐dependent endocytosis were adopted to further analyse the interaction between two routes. According to a previous publication (Zhou, Jiang, et al. 2023; Zhou, Wang, et al. 2023), mycelial discs were cultured on PDA medium containing ddH_2_O, 0.2 μM CPZ, DMSO or 0.2 μM Baf A, and harvested at 6, 11 and 16 days after culture. The relative expression levels of the above four genes related to transmembrane transport were examined using qRT‐PCR. Meanwhile, the mycelia were harvested at 6 and 16 days after culture and incubated with 50 μL TPE‐SPc solution (20 ng/μL) for 30 min. The uptake efficiency of TPE‐SPc by mycelia was observed using the inverted fluorescence microscope (EVOS FL).

To further confirm the interactional relationship of two routes, the mycelial mutant for *pkc* or *mfs3* was constructed according to a previous method (Müller et al. 2018). More specifically, the mycelial discs were cultured on PDA medium in the dark for 60 h. For the preparation of protoplasts, the mycelia were harvested and then incubated with enzymatic solution (0.1% Snailase and 1% lysozyme) in the dark at 28°C, 120 rpm for 3 h. The enzymatic solution was filtered through a sterile nylon cloth (25–30 μm pore size) and the protoplasts were harvested, washed with 500 μL KCl and CaCl_2_ buffer, and resuspended in TMSC buffer (1 M D‐sorbitol, 10 mM MOPS, 50 mM CaCl_2_, pH 6.3). For transformation, 6 μg Cas9‐NLS protein, 2 μg sgRNA and 0.4 μL 10× buffer were incubated at 37°C for 30 min. Then, 10 μg repair template, 60 μL Tris‐CaCl_2_ buffer (10 mM Tris–HCl, 1 mM EDTA, 40 mM CaCl_2_, pH 6.3) and 100 μL protoplast solution were incubated on ice for 5 min. Next, 160 μL PEG solution (1 M sorbitol, 10 mM MOPS, 0.6 g/mL PEG3350) was added, and the mixture was incubated for 20 min. Then, 680 μL TMSC buffer was added and centrifuged to remove the supernatant. The protoplasts were resuspended in 200 μL TMSC buffer, spread on the SH agar plates (0.6 M sucrose, 5 mM Tris–HCl, 1 mM NH_4_H_2_PO_4_, 8 g/L agar, 17.5 μg/mL hygromycin B) and cultured for 3 days. The positive colonies were selected on malt extract medium (10 g malt extract, 4 g glucose, 4 g yeast extract, 10 g agar, made up to 1 L with water, 35 μg/mL hygromycin B) for 3–5 generations. The expression of above seven genes in two routes was examined using the qRT‐PCR, and the uptake efficiency of TPE‐SPc by mycelial mutants was then observed using the inverted fluorescence microscope (EVOS FL).

### 3.12 Preparation of RNA Fungicides for Grey Mould Disease

The *dcl1* and *dcl2* are responsible for the production of fungal siRNA effectors, and their RNAi can inhibit the plant immune system and facilitate the infection of grey mould disease (Wang et al. 2016). Additionally, the *GAP1* gene is crucial for the development and virulence of *B. cinerea* and *Sclerotinia sclerotiorum* (Xu et al. 2024). The *dcl1* (XM_024696562.1), *dcl2* (XM_024697207.1) and *iqg1* (XM_001555933.2, a homologous sequence of *Sclerotinia sclerotiorum GAP1* gene) were used as target genes for the preparation of RNA fungicides with the aid of SPc. The prepared RNA fungicides included the ds*dcl1*/SPc complexes, ds*dcl2*/SPc complexes, ds*iqg1*/SPc complexes, ds*dcl1* + ds*iqg1*/SPc complexes and ds*dcl2* + ds*iqg1*/SPc complexes. The corresponding RNA fungicides without SPc were also prepared and employed as controls.

### 3.13 Wettability and Retention Analysis of RNA Fungicides on Cucumber Leaves

Cucumber leaves were immobilized on microscope slides, and 6 μL above SPc‐based RNA fungicides (dsRNA concentration: 100 ng/μL) were individually applied to the leaf surface with a micro syringe. The contact angles were examined using the Optical Contact Angle Meter (Date Physics Corporation OCA25, Germany). A commercial fungicide 1.5% osthole‐matrine (Shanxi Dewei Herbal Biotechnology Co., China) was diluted to 400 times with ddH_2_O, and its contact angle was also measured. The ddH_2_O and SPc were employed as controls.

Retention of droplets is a key factor influencing the deposition of active ingredients on leaf surfaces. Fresh cucumber leaves (4 cm^2^) were weighed (*M*_1_), immersed in SPc‐based RNA fungicides (dsRNA concentration: 100 ng/μL) for 15 s, lifted vertically with tweezers until no liquid remained dripping, and weighed (*M*_2_). The 1.5% osthole‐matrine (Shanxi Dewei Herbal Biotechnology Co.), ddH_2_O and SPc were also tested. Retention (mg/cm^2^) was calculated using the following formula:

(3) $$\text{Retention}\;\left(\mathrm{mg}/{\mathrm{cm}}^{2}\right)=\left(M_{2}–M_{1}\right)÷\text{leaf area}$$

### 3.14 Protective Effect Assay of RNA Fungicides Toward Grey Mould Disease

The protective effects of RNA fungicides with/without SPc on grey mould disease were evaluated according to a previous publication (Islam et al. 2021). Various RNA fungicides (dsRNA concentration: 1000 ng/mL, 1 mL) were sprayed on the leaves and fruits, respectively. The 1.5% osthole‐matrine (Shanxi Dewei Herbal Biotechnology Co.) diluted to 400 times with ddH_2_O was applied as positive control, and ddH_2_O and SPc were employed as negative controls. The treated leaves or fruits were then inoculated with 5 mm *B. cinerea* mycelium, and the images were captured at 3, 4 and 6 days after the inoculation. The images of untreated leaves and fruits were also captured. The relative lesion sizes were measured using the ImageJ 1.8 software.

### 3.15 Statistical Analysis

Data analysis was performed using the Prism 8.3.0 software (GraphPad Software Inc., USA). The significance level was set at *p* < 0.05. The descriptive statistics are shown as the mean value and standard errors of the mean.

## Author Contributions

J.S., S.Y. and M.G. designed the project and interpreted the data. M.G. performed most of the experiments. M.G., C.X., Y.X., J.L., Q.J., S.Y. and J.S. analysed the data. M.Y. provided nanomaterial resources. S.Y. and M.G. wrote the manuscript. All authors edited the manuscript.

## Funding

This work was supported by the Joint Research Program of State Key Laboratory of Agricultural and Forestry Biosecurity (No. SKLJRP2506), National Key Research and Development Program of China (2023YFD1700304, 2024YFC2607600), 2115 Talent Development Program of China Agricultural University and China Agricultural University Graduate Student Independent Innovation Research Fund Project (2024TC180).

## Conflicts of Interest

The authors declare no conflicts of interest.

## Supporting information


**Figure S1:** Enhanced stability of SPc‐loaded dsRNA treated with RNase A. (a) Complexation with SPc prevented the ds*eGFP* migration to the positive electrode. To assess the protective effect of SPc on ds*eGFP* (420 bp), electrophoresis was conducted after the incubation of RNase A with naked ds*eGFP* or ds*eGFP*/SPc complex for 20 min. The 0.5% SDS was added to release ds*eGFP* from the ds*eGFP*/SPc complex. (b) Relative ds*eGFP* amount was analysed using the Image J 1.8 software (*n* = 3 replications). Different letters above each bar indicate significant differences at *p* < 0.05 as determined by one‐way ANOVA with Tukey HSD test (*F*_3,8_ = 166.3, *p* < 0.0001). (c) Complexation with SPc prevented the ds*tublin* migration to the positive electrode. To assess the protective effect of SPc on ds*tublin* (1047 bp), electrophoresis was conducted after the incubation of RNase A with naked ds*tublin* or ds*tublin*/SPc complex for 20 min. The 0.5% SDS was added to release ds*tublin* from the ds*tublin*/SPc complex. (d) Relative ds*tublin* amount was analysed using the Image J 1.8 software (*n* = 3 replications). Different letters above each bar indicate significant differences at *p* < 0.05 as determined by one‐way ANOVA with Tukey HSD test (*F*_3,8_ = 13.76, *p* = 0.0016). Bar represents mean ± SEM.
**Figure S2:** Standard curve for quantifying ds*eGFP* using the qRT‐PCR. The Ct numbers of naked ds*eGFP* with various qualities were determined.
**Figure S3:** Pearson correlation between collected samples for RNA‐seq analysis.
**Figure S4:** Overlapping DEGs between collected samples for RNA‐seq analysis. (a) Analysis of DEGs with Venn diagram. (b) KEGG enrichment of overlapping DEGs. (c) Heat maps of overlapping DEGs associated with endocytosis and transmembrane transport. Genes with high expression levels are shown in red, while those with low expression levels appear in white.
**Figure S5:** Schematic diagram for main transport‐related pathways. (a) Endocytic pathways (Clathrin‐dependent and independent endocytosis). Up‐regulated genes are shown in red. (b) Transmembrane transport‐mediated delivery (MFS transporter and ABC transporter). The *mfs2* and *mfs4* contained MFS domain, but they were not annotated in the KEGG enrichment.
**Figure S6:** Visualization of SPc‐mediated dsRNA delivery enhancement via two routes in *B. cinerea* . (a) Representative TEM images of ds*eGFP* delivery by SPc via two routes endocytosis and transmembrane transport. (b) SPc‐mediated activation of two routes for enhanced dsRNA delivery. In route 1, the number and area of vesicles in the mycelia incubated with ds*eGFP* and ds*eGFP*/SPc complex were quantified. Each treatment was repeated seven times. The asterisk indicates significant difference according to independent *t*‐test (two‐tailed, ***p < 0.001, **p < 0.01). Vesicle number (*t* = 4.38, df = 12, *p* = 0.0009) and relative vesicle area (*t* = 3.07, df = 12, *p* = 0.0097). In route 2, the particle number in the mycelia incubated with ds*eGFP* and ds*eGFP*/SPc complex was recorded. Each treatment consisted of five independent images. The asterisk indicates significant difference according to independent *t*‐test (two‐tailed, *t* = 2.34, df = 8, *p* = 0.0473). Bar represents mean ± SEM.
**Figure S7:** sgRNA screening and mutant phenotype. (a) sgRNA screening in vitro. M, DNA marker. (b) Phenotypes of *pkc* and *mfs3* mutant strains.
**Figure S8:** Expression levels of *dcl1*, *dcl2* and *iqg1* in *B. cinerea* incubated with dsRNA/SPc complex using the qRT‐PCR. Each treatment consisted of three replications. The asterisk indicates significant difference according to independent *t*‐test (two‐tailed, *p < 0.05, **p < 0.01 and ***p < 0.001). *dcl1* (3 days: *t* = 3.17, df = 4, *p* = 0.0338; 4 days: *t* = 3.51, df = 4, *p* = 0.0247), *dcl2* (3 days: *t* = 6.03, df = 4, *p* = 0.0038; 4 days: *t* = 6.20, df = 4, *p* = 0.0034) and *iqg1* (3 days: *t* = 8.56, df = 4, *p* = 0.0010; 4 days: *t* = 15.40, df = 4, *p* = 0.0001). Bar represents mean ± SEM.
**Figure S9:** Protective effects of naked dsRNA toward grey mould disease. (a) Protective effects of dsRNA on cucumber leaves. Incisions were made on the leaves, and various formulations were sprayed on the leaves, which were then inoculated with *B. cinerea* mycelium. The images were captured at 3, 4 and 6 days after the inoculation, and the lesion sizes were measured (*n* = 3 replications). Different letters above each bar indicate significant differences at *p* < 0.05 as determined by one‐way ANOVA with Tukey HSD test (3 days: *F*_5,12_ = 0.71, *p* = 0.6248; 4 days: *F*_5,12_ = 0.56, *p* = 0.7267; 6 days: *F*_5,12_ = 0.70, *p* = 0.6331). (b) Protective effects of dsRNA on tomato fruits. Holes were made in the centre of fruits, and various formulations were sprayed on the fruits, which were then inoculated with *B. cinerea* mycelium. Images were captured at 3, 4 and 6 days after the inoculation, and the lesion sizes were measured (*n* = 3 replications). Different letters above each bar indicate significant differences at *p* < 0.05 as determined by one‐way ANOVA with Tukey HSD test (3 days: *F*_5,12_ = 0.07, *p* = 0.9953; 4 days: *F*_5,12_ = 1.18, *p* = 0.3716; 6 days: *F*_5,12_ = 0.48, *p* = 0.7829). (a, b) Bar represents mean ± SEM.
**Table S1:** Sequencing quality for RNA‐seq analysis.
**Table S2:** Key DEGs related to endocytosis and transmembrane transport identified from RNA‐seq analysis.
**Table S3:** Primers used in this study.
**Table S4:** Hygromycin resistance sequence used in this study.

## Data Availability

The data that support the findings of this study are available in the Supporting Information of this article.
