# Supplementary material for: Interactional Endocytosis and Transmembrane Transport Promote Cellular Internalization of Nano‐Delivered RNA Drugs for Efficient Control of Crop Diseases
Source: Plant Biotechnol J. 2026 May 29;24(9):5152–71. doi: 10.1111/pbi.70689 (PMC13398849; doi:10.1111/pbi.70689)
Supplement: Supplementary file 1 [file PBI-24-5152-s001.zip › pbi70689-sup-0001-Supinfo.docx]

Supporting Information

**Interactional endocytosis and transmembrane transport promote cellular internalization of nano-delivered RNA drugs for efficient control of crop diseases**

**Mei Guan^1^, Chao Xie^1^, Yang Xue^1^, Juan Lu^1^, Qinhong Jiang^1^, Meizhen Yin^2^, Shuo Yan^1🖂^ & Jie Shen^1🖂^**

^1^State Key Laboratory of Agricultural and Forestry Biosecurity, MARA Key Lab of Surveillance and Management for Plant Quarantine Pests, College of Plant Protection, China Agricultural University, Beijing 100193, China. ^2^State Key Laboratory of Chemical Resource Engineering, Beijing University of Chemical Technology, Beijing 100029, China. 🖂e-mail: shenjie@cau.edu.cn; yanshuo2011@foxmail.com

**Figure S1. Enhanced stability of SPc-loaded dsRNA treated with RNase A.** **a** Complexation with SPc prevented the ds*eGFP* migration to the positive electrode. To assess the protective effect of SPc on ds*eGFP* (420 bp), electrophoresis was conducted after the incubation of RNase A with naked ds*eGFP* or ds*eGFP*/SPc complex for 20 min. The 0.5% SDS was added to release ds*eGFP* from the ds*eGFP*/SPc complex. **b** Relative ds*eGFP* amount was analyzed using the Image J 1.8 software (*n* = three replications). Different letters above each bar indicate significant differences at *p* < 0.05 as determined by one-way ANOVA with Tukey HSD test (*F_3,8_* = 166.3, *p* < 0.0001). **c** Complexation with SPc prevented the ds*tublin* migration to the positive electrode. To assess the protective effect of SPc on ds*tublin* (1047 bp), electrophoresis was conducted after the incubation of RNase A with naked ds*tublin* or ds*tublin*/SPc complex for 20 min. The 0.5% SDS was added to release ds*tublin* from the ds*tublin*/SPc complex. **d** Relative ds*tublin* amount was analyzed using the Image J 1.8 software (*n* = three replications). Different letters above each bar indicate significant differences at *p* < 0.05 as determined by one-way ANOVA with Tukey HSD test (*F_3,8_* = 13.76, *p* = 0.0016). Bar represents mean ± SEM.

**Figure S2. Standard curve for quantifying ds*eGFP* using the qRT-PCR.** The Ct numbers of naked ds*eGFP* with various qualities were determined.

**Figure S3.** **Pearson correlation between collected samples for RNA-seq analysis.**

**Figure S4.** **Overlapping DEGs between collected samples for RNA-seq analysis. a** Analysis of DEGs with venn diagram. **b** KEGG enrichment of overlapping DEGs. **c** Heat maps of overlapping DEGs associated with endocytosis and transmembrane transport. Genes with high expression levels are shown in red, while those with low expression levels appear in white.

**Figure S5.** **Schematic diagram for main transport-related pathways.** **a** Endocytic pathways (Clathrin-dependent and independent endocytosis). Up-regulated genes are shown in red. **b** Transmembrane transport-mediated delivery (MFS transporter and ABC transporter). The *mfs2* and *mfs4* contained MFS domain, but they were not annotated in the KEGG enrichment.

**Figure S6.** **Visualization of SPc-mediated dsRNA delivery enhancement via two routes in *B. cinerea*. a** Representative TEM images of ds*eGFP* delivery by SPc via two routes endocytosis and transmembrane transport. **b** SPc-mediated activation of two routes for enhanced dsRNA delivery. In route 1, the number and area of vesicles in the mycelia incubated with ds*eGFP* and ds*eGFP*/SPc complex were quantified. Each treatment was repeated seven times. The asterisk indicates significant difference according to independent *t* test (Two-tailed, ****p* < 0.001, ***p* < 0.01). Vesicle number (*t* = 4.38, df = 12, *p* = 0.0009*)*, and relative vesicle area (*t* = 3.07, df = 12, *p* = 0.0097). In route 2, the particle number in the mycelia incubated with ds*eGFP* and ds*eGFP*/SPc complex was recorded. Each treatment consisted of five independent images. The asterisk indicates significant difference according to independent *t* test (Two-tailed, *t* = 2.34, df = 8, *p* =0.0473). Bar represents mean ± SEM.

**Figure S7.** **sgRNA screening and mutant phenotype. a** sgRNA screening *in vitro*. M: DNA marker. **b** Phenotypes of *pkc* and *mfs3* mutant strains.

**Figure S8.** **Expression levels of *dcl1*, *dcl2* and *iqg1* in *B. cinerea* incubated with dsRNA/SPc complex using the qRT-PCR.** Each treatment consisted of three replications. The asterisk indicates significant difference according to independent *t* test (Two-tailed, **p* < 0.05, ***p* < 0.01 and ****p* < 0.001). *dcl1* (3 d: *t* = 3.17, df = 4, *p* = 0.0338; 4 d: *t* = 3.51, df = 4, *p* = 0.0247), *dcl2* (3 d: *t* = 6.03, df = 4, *p* = 0.0038; 4 d: *t* =6.20, df = 4, *p* = 0.0034) and *iqg1* (3 d: *t* = 8.56, df = 4, *p* = 0.0010; 4 d: *t* = 15.40, df = 4, *p* = 0.0001). Bar represents mean ± SEM.

**Figure S9. Protective effects of naked dsRNA toward gray mold disease. a** Protective effects of dsRNA on cucumber leaves. Incisions were made on the leaves, and various formulations were sprayed on the leaves, which were then inoculated with *B. cinerea* mycelium. The images were captured at 3, 4 and 6 d after the inoculation, and the lesion sizes were measured (*n* = three replications). Different letters above each bar indicate significant differences at *p* < 0.05 as determined by one-way ANOVA with Tukey HSD test (3 d: *F_5,12_* = 0.71, *p* = 0.6248; 4 d: *F_5,12_* = 0.56, *p* = 0.7267; 6 d: *F_5,12_* = 0.70, *p* = 0.6331). **b** Protective effects of dsRNA on tomato fruits. Holes were made in the center of fruits, and various formulations were sprayed on the fruits, which were then inoculated with *B. cinerea* mycelium. Images were captured at 3, 4 and 6 d after the inoculation, and the lesion sizes were measured (*n* = three replications). Different letters above each bar indicate significant differences at *p* < 0.05 as determined by one-way ANOVA with Tukey HSD test (3 d: *F_5,12_* = 0.07, *p* = 0.9953; 4 d: *F_5,12_* = 1.18, *p* = 0.3716; 6 d: *F_5,12_* = 0.48, *p* = 0.7829). **a**-**b** Bar represents mean ± SEM*.*

**Table S1. Sequencing quality for RNA-seq analysis.**

| Sample | Clean read | Clean base | GC content | Q30 |
| --- | --- | --- | --- | --- |
| ddH_2_O-1 | 2099342 | 6,283,418,688 | 46.98% | 93.33% |
| ddH_2_O-2 | 20,275,366 | 6,069,758,695 | 46.96% | 92.91% |
| ddH_2_O-3 | 20,753,912 | 6,213,277,867 | 46.92% | 93.40% |
| SPc-1 | 20,802,162 | 6,227,921,280 | 46.94% | 93.10% |
| SPc-2 | 20,650,891 | 6,183,187,828 | 46.95% | 93.64% |
| SPc-3 | 20,416,253 | 6,115,819,126 | 47.03% | 92.87% |
| ds*eGFP*-1 | 21290429 | 6376131144 | 47.09% | 93.23% |
| ds*eGFP*-2 | 24780051 | 7420736294 | 47.06% | 93.84% |
| ds*eGFP*-3 | 25816905 | 7731179880 | 47.03% | 93.31% |
| ds*eGFP*/SPc-1 | 23881947 | 7140905730 | 46.82% | 93.48% |
| ds*eGFP*/SPc-2 | 23613920 | 7071160770 | 46.90% | 93.09% |
| ds*eGFP*/SPc-3 | 26328993 | 7884743874 | 46.96% | 93.42% |

**Table S2. Key DEGs related to endocytosis and transmembrane transport identified from RNA-seq analysis.**

| **Route** | **Gene symbol** | **Abbreviation** |
| --- | --- | --- |
| Endocytosis | *BCIN_13g03260* | *pkc* |
|  | *Bcypt10* | *ypt10* |
|  | *BCIN_11g05390* | *pil1* |
| Transmembrane transport | *BCIN_09g05240* | *mfs1* |
|  | *BCIN_06g01490* | *mfs2* |
|  | *BCIN_06g04860* | *mfs3* |
|  | *BCIN_09g05940* | *mfs4* |

**Table S3. Primers used in this study.**

| **Primer** | **Sequence (5’-3’)** | **Application** |
| --- | --- | --- |
| *eGFP* | TAATACGACTCACTATAGGCACAAGTTCAGCGTGTCCG  TAATACGACTCACTATAGGGTTCACCTTGATGCCGTTC | dsRNA synthesis |
| *tublin* | TAATACGACTCACTATAGGCAACTCTTCCGTCCCGACAA  TAATACGACTCACTATAGGAGAGATCGAGGCATCCTGGT |  |
| *pkc* | TAATACGACTCACTATAGGGGAGCGCTCAAAAGGAGGAT  TAATACGACTCACTATAGGAGTCCGAGAGCATAAAGCCG |  |
| *ypt10* | TAATACGACTCACTATAGGGCCGATCTCGTCGCTAAAGA  TAATACGACTCACTATAGGACCGCGCTCAAAAGTTGTTC |  |
| *pil1* | TAATACGACTCACTATAGGTCCCTCTCTTCCCTTCGTGG  TAATACGACTCACTATAGGAGCACTTTGAGGCTCCTTGG |  |
| *mfs1* | TAATACGACTCACTATAGGAGTGCGGAAATCATCCAAAG  TTAATACGACTCACTATAGGTTTGTCCAGTGGTGACGGTA |  |
| *mfs2* | TAATACGACTCACTATAGGGCATTCGGTTGTGAATTGTG  TAATACGACTCACTATAGGCAAGCCAGATTAGCCTCGAC |  |
| *mfs3* | TAATACGACTCACTATAGGGGTTCTTGGGTATGGCTGAA  TAATACGACTCACTATAGGGCCCAGCCACATCTTGTAAT |  |
| *mfs4* | TAATACGACTCACTATAGGTATCGTCCTTCAATCTGGGC  TAATACGACTCACTATAGGACGAAAGCCAGTCCAGCTAA |  |
| *dcl1* | TTAATACGACTCACTATAGGTGCGGAAGAACTTGAAGGT  TTGCTACA  TAATACGACTCACTATAGGGCAGCAAATGGCATCCGTCCAGATCTGGTCAACACACCAAG |  |
| *dcl2* | TAATACGACTCACTATAGGCTTGGTGTGTTGACCAGATCTGGACGGATGCCATTTGCTGC  TAATACGACTCACTATAGGACTCTTGAGTACTTTCGCCAGCTCAC |  |
| *iqg1* | TAATACGACTCACTATAGGAGGGGAGTAACAGCAGAGCA  TAATACGACTCACTATAGGAGTTCGGCCGTGTGTTTATC |  |
| *q*-*actin* | CTGGAATGTGTAAGGCCGGT  ACCGTGCTCGATTGGGTATC | qRT-PCR |
| *q*-*pkc* | GGGTGTACCTCGCGGATTTT  GGAGCTATCCAAGACGCAGT |  |
| *q*-*ypt10* | GCACCTCCCTCGAAGCTAAG  TCAGAAAACTCGCACCCACA |  |
| *q*-*pil1* | ACACCAATCGTGCCAGGAAA  GCGATTGAACTTCTTCGGCAT |  |
| *q*-*mfs1* | GAGCAAGACCAGCGCTAAGA  CGAGCAGGAGAATGAGCACT |  |
| *q*-*mfs2* | ACCGCAAAGTACGCATGAGA  CACCGCTGCATGATTTGCTT |  |
| *q*-*mfs3* | AATGCGAAGATCGATGGCCT  GGCGAAGGCGTTTCAAAAGA |  |
| *q*-*mfs4* | ACACATCGTTGCCTGGAAGT  AAGTGATCGTTCTGGCGAGG |  |
| *q*-*dcl1* | TGGGCAAAGGCTAGAGCAAA  AGTCTCGAGCTCACCTCCAT |  |
| *q*-*dcl2* | TGCCAAGGCTCTCAGACAAG  CGGAAAAGGGAGGCTTCTGT |  |
| *q*-*iqg1* | TTGGAAGATGCGACCGACTT  TGCCTGGGCTCTGATTGAAG |  |
| sg*pkc*-1-F | AAGCTAATACGACTCACTATAGGAGGAAGCTAATGCCATGGGTTTTAGAGCTAGAAATAGC | Transformation |
| sg*pkc*-2-F | AAGCTAATACGACTCACTATAGATTCGTTCCGATACGTGCGGTTTTAGAGCTAGAAATAGC |  |
| 60 bp-F-*pkc* | TACATATATTCTTACACTTAATTGTACAGTAAGATTTCGTTCATTCAAAAAACCTTTCCAGACTGCCTCAGGTGGGGC |  |
| 60 bp-R-*pkc* | CAAGTTCTAGAATATCCGAATAATAGATCATGGGTGTCATGTAGTTTTGCTCGAAGTTTTTAATGCATTGCAGATGAGCTGTATC |  |
| F-*pkc* | GGATGCCAGCCACGAACT |  |
| R-*pkc* | CAACTCTGACGACACGCAAAG |  |
| sg*mfs3*-1-F | AAGCTAATACGACTCACTATAGAGAGGAAGTAGTTTACGCCGTTTTAGAGCTAGAAATAGC |  |
| sg*mfs3*-2-F | AAGCTAATACGACTCACTATAGGCATGGATCTTCATCATTGGTTTTAGAGCTAGAAATAGC |  |
| sgRNA-scaffold-R | AAAAGCACCGACTCGGTGCCACTTTTTCAAGTTGATAACGGACTAGCCTTATTTTAACTTGCTATTTCTAGCTCTAAAAC |  |
| 60 bp-F-*mfs3* | TAGCACAGAGAAGGAAATCGAAATTCGACTGCATAATTCTATAGTTACACCTGTCTAATCGACTGCCTCAGGTGGGGC |  |
| 60 bp-R-*mfs3* | CAGTAAAAGCGAGTAGAAGCTTGTTAACTCACAGTTTGCATATGACACTTAACGTTGACTTAATGCATTGCAGATGAGCTGTATC |  |
| F-*mfs3* | ATACCTGCATCGACGCAAAG |  |
| R-*mfs3* | AGCCAAGTAAACGGGTCAAA |  |

The T7 promoter sequence is underlined.

**Table S4. Hygromycin resistance sequence used in this study.**

| Pcpc1-HygR-Ttrpc | gactgcctcaggtggggcagtgctagtgtgtgtaccgacccgcaggattggtgctttgcccagagctctacagaatagcgcgcgcatccatatgttagttctgcaattttcttgtatcggtgctgtgactcatacttccccctttggctggccttgcggcaaccaataagaacgcacagtgaaatcttgcgggtggggagtggatccatggcgcctgcattggcttggggacgcgcactgtcgcacacttccatctgacctttcagaagggtttcgtggtgggcaaggaccaaccggttgcgcggccgtgcgtgggtgcctcgcccggcactgccagggccactgcagtggcagtttgctgcctgatacaaaatccttccctccgcccagttttccctctttgaccttcctttcttcttctctgcaaccaaatccaccctatcaaaccaaaacagtatctcgaccgaggtatcaacctgaatcagcaacatcgtagccagcatttgtctccgtctctgcagaaccagcgagttgcaaacattatccaggcaacagggcaccaactcacttcttcggctttcaccaatcggtacagctcttctcagaactcgcgtccgcaacagttctacgcttcctcagcaccttcttcagcttcaatcctgaacactcagaaccgcgcacagcagcgccctcctgttcccttgtttcccaaaagtaccggtagtatttcgcacggaaagcagggcaacaagatgttctcaggtacccatatgaaaaagcctgaactcaccgcgacgtctgtcgagaagtttctgatcgaaaagttcgacagcgtctccgacctgatgcagctctcggagggcgaagaatctcgtgctttcagcttcgatgtaggagggcgtggatatgtcctgcgggtaaatagctgcgccgatggtttctacaaagatcgttatgtttatcggcactttgcatcggccgcgctcccgattccggaagtgcttgacattggggaattcagcgagagcctgacctattgcatctcccgccgtgcacagggtgtcacgttgcaagacctgcctgaaaccgaactgcccgctgttctgcagccggtcgcggaggccatggatgcgatcgctgcggccgatcttagccagacgagcgggttcggcccattcggaccgcaaggaatcggtcaatacactacatggcgtgatttcatatgcgcgattgctgatccccatgtgtatcactggcaaactgtgatggacgacaccgtcagtgcgtccgtcgcgcaggctctcgatgagctgatgctttgggccgaggactgccccgaagtccggcacctcgtgcacgcggatttcggctccaacaatgtcctgacggacaatggccgcataacagcggtcattgactggagcgaggcgatgttcggggattcccaatacgaggtcgccaacatcttcttctggaggccgtggttggcttgtatggagcagcagacgcgctacttcgagcggaggcatccggagcttgcaggatcgccgcgcctccgggcgtatatgctccgcattggtcttgaccaactctatcagagcttggttgacggcaatttcgatgatgcagcttgggcgcagggtcgatgcgacgcaatcgtccgatccggagccgggactgtcgggcgtacacaaatcgcccgcagaagcgcggccgtctggaccgatggctgtgtagaagtactcgccgatagtggaaaccgacgccccagcactcgtccgagggcaaaggaatagagtagatgccgaccgggatccacttaacgttactgaaatcatcaaacagcttgacgaatctggatataagatcgttggtgtcgatgtcagctccggagttgagacaaatggtgttcaggatctcgataagatacgttcatttgtccaagcagcaaagagtgccttctagtgatttaatagctccatgtcaacaagaataaaacgcgtttcgggtttacctcttccagatacagctcatctgcaatgcatta |
| --- | --- |

The sequences of Pcpc1, HygR and Ttrpc are shown in blue, green and red, respectively.
