# Supplementary material for: Interactional Endocytosis and Transmembrane Transport Promote Cellular Internalization of Nano‐Delivered RNA Drugs for Efficient Control of Crop Diseases
Source: Plant Biotechnol J. 2026 May 29:10.1111/pbi.70689. Online ahead of print. doi: 10.1111/pbi.70689 (PMC13398849; doi:10.1111/pbi.70689)
Supplement: Supplementary file 1 — Figure S1: Enhanced stability of SPc‐loaded dsRNA treated with RNase A. (a) Complexation with SPc prevented the dseGFP migration to the positive electrode. To assess the protective effect of SPc on dseGFP (420 bp), electrophoresis was conducted after the incubation of RNase A with naked dseGFP or dseGFP/SPc complex for 20 min. The 0.5% SDS was added to release dseGFP from the dseGFP/SPc complex. (b) Relative dseGFP amount was analysed using the Image J 1.8 software (n = 3 replications). Different letters above each bar indicate significant differences at p < 0.05 as determined by one‐way ANOVA with Tukey HSD test (F 3,8 = 166.3, p < 0.0001). (c) Complexation with SPc prevented the dstublin migration to the positive electrode. To assess the protective effect of SPc on dstublin (1047 bp), electrophoresis was conducted after the incubation of RNase A with naked dstublin or dstublin/SPc complex for 20 min. The 0.5% SDS was added to release dstublin from the dstublin/SPc complex. (d) Relative dstublin amount was analysed using the Image J 1.8 software (n = 3 replications). Different letters above each bar indicate significant differences at p < 0.05 as determined by one‐way ANOVA with Tukey HSD test (F 3,8 = 13.76, p = 0.0016). Bar represents mean ± SEM. Figure S2: Standard curve for quantifying dseGFP using the qRT‐PCR. The Ct numbers of naked dseGFP with various qualities were determined. Figure S3: Pearson correlation between collected samples for RNA‐seq analysis. Figure S4: Overlapping DEGs between collected samples for RNA‐seq analysis. (a) Analysis of DEGs with Venn diagram. (b) KEGG enrichment of overlapping DEGs. (c) Heat maps of overlapping DEGs associated with endocytosis and transmembrane transport. Genes with high expression levels are shown in red, while those with low expression levels appear in white. Figure S5: Schematic diagram for main transport‐related pathways. (a) Endocytic pathways (Clathrin‐dependent and independent endocytosis). Up‐regulated gene [file PBI-9999-0-s001.zip › pbi70689-sup-0001-Supinfo.docx]

Supporting Information

**Interactional endocytosis and transmembrane transport promote cellular internalization of nano-delivered RNA drugs for efficient control of crop diseases**

**Mei Guan^1^, Chao Xie^1^, Yang Xue^1^, Juan Lu^1^, Qinhong Jiang^1^, Meizhen Yin^2^, Shuo Yan^1🖂^ & Jie Shen^1🖂^**

^1^State Key Laboratory of Agricultural and Forestry Biosecurity, MARA Key Lab of Surveillance and Management for Plant Quarantine Pests, College of Plant Protection, China Agricultural University, Beijing 100193, China. ^2^State Key Laboratory of Chemical Resource Engineering, Beijing University of Chemical Technology, Beijing 100029, China. 🖂e-mail: shenjie@cau.edu.cn; yanshuo2011@foxmail.com


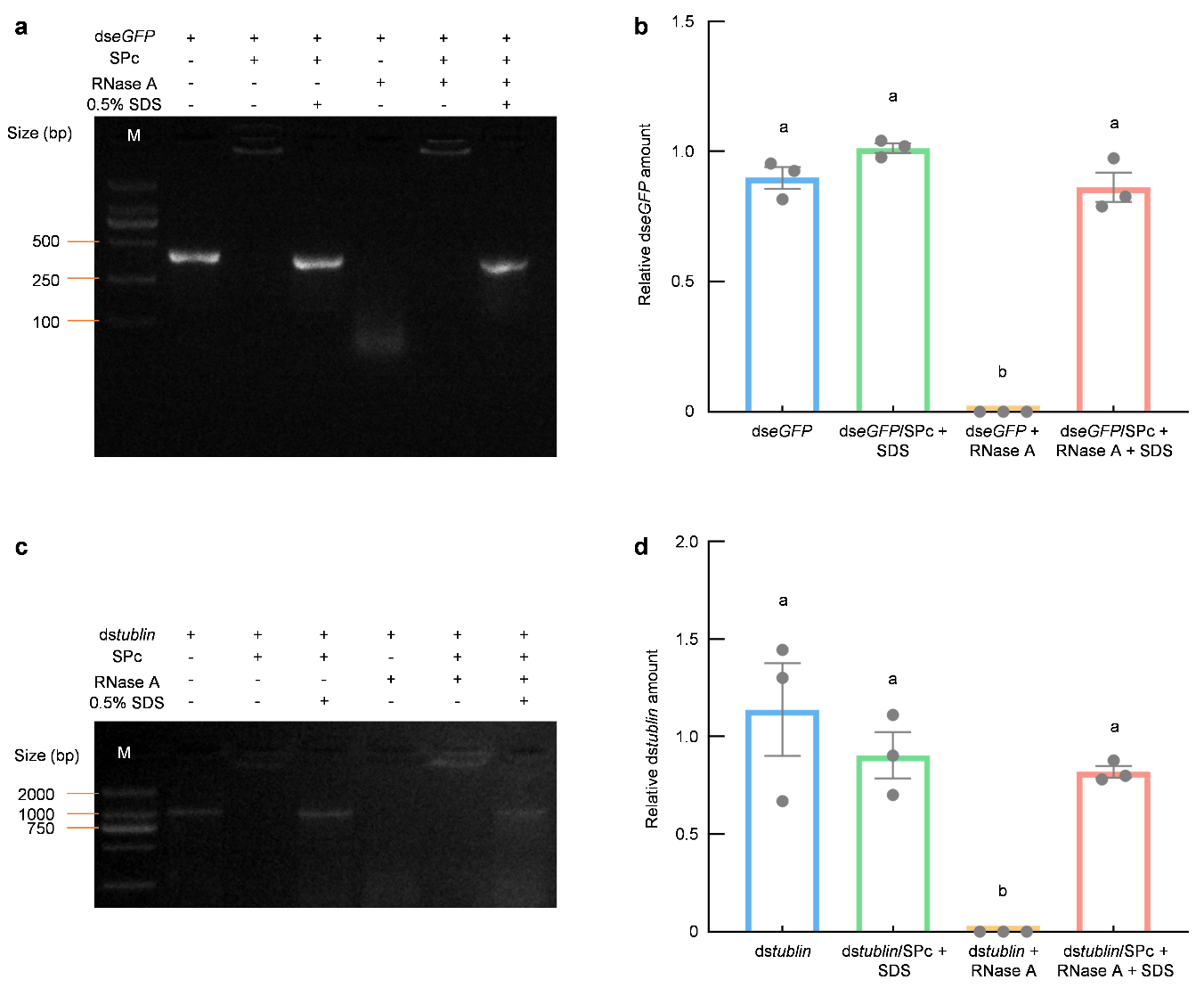


**Figure S1. Enhanced stability of SPc-loaded dsRNA treated with RNase A.** **a** Complexation with SPc prevented the ds*eGFP* migration to the positive electrode. To assess the protective effect of SPc on ds*eGFP* (420 bp), electrophoresis was conducted after the incubation of RNase A with naked ds*eGFP* or ds*eGFP*/SPc complex for 20 min. The 0.5% SDS was added to release ds*eGFP* from the ds*eGFP*/SPc complex. **b** Relative ds*eGFP* amount was analyzed using the Image J 1.8 software (*n* = three replications). Different letters above each bar indicate significant differences at *p* < 0.05 as determined by one-way ANOVA with Tukey HSD test (*F_3,8_* = 166.3, *p* < 0.0001). **c** Complexation with SPc prevented the ds*tublin* migration to the positive electrode. To assess the protective effect of SPc on ds*tublin* (1047 bp), electrophoresis was conducted after the incubation of RNase A with naked ds*tublin* or ds*tublin*/SPc complex for 20 min. The 0.5% SDS was added to release ds*tublin* from the ds*tublin*/SPc complex. **d** Relative ds*tublin* amount was analyzed using the Image J 1.8 software (*n* = three replications). Different letters above each bar indicate significant differences at *p* < 0.05 as determined by one-way ANOVA with Tukey HSD test (*F_3,8_* = 13.76, *p* = 0.0016). Bar represents mean ± SEM.


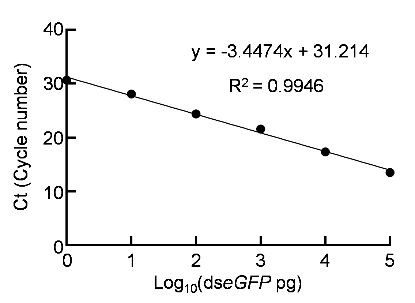


**Figure S2. Standard curve for quantifying ds*eGFP* using the qRT-PCR.** The Ct numbers of naked ds*eGFP* with various qualities were determined.


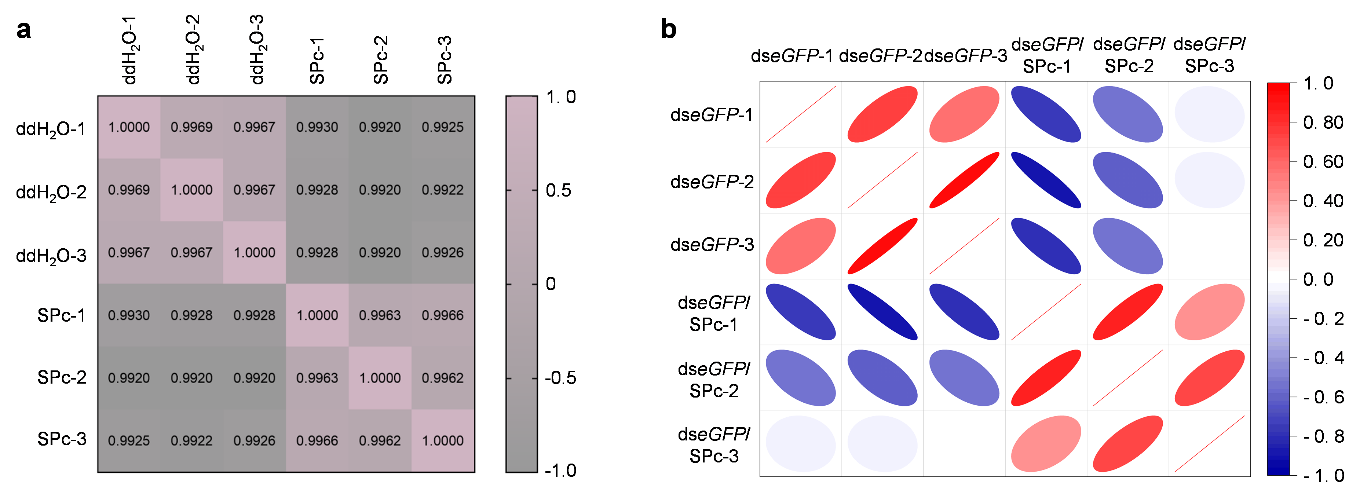


**Figure S3.** **Pearson correlation between collected samples for RNA-seq analysis.**


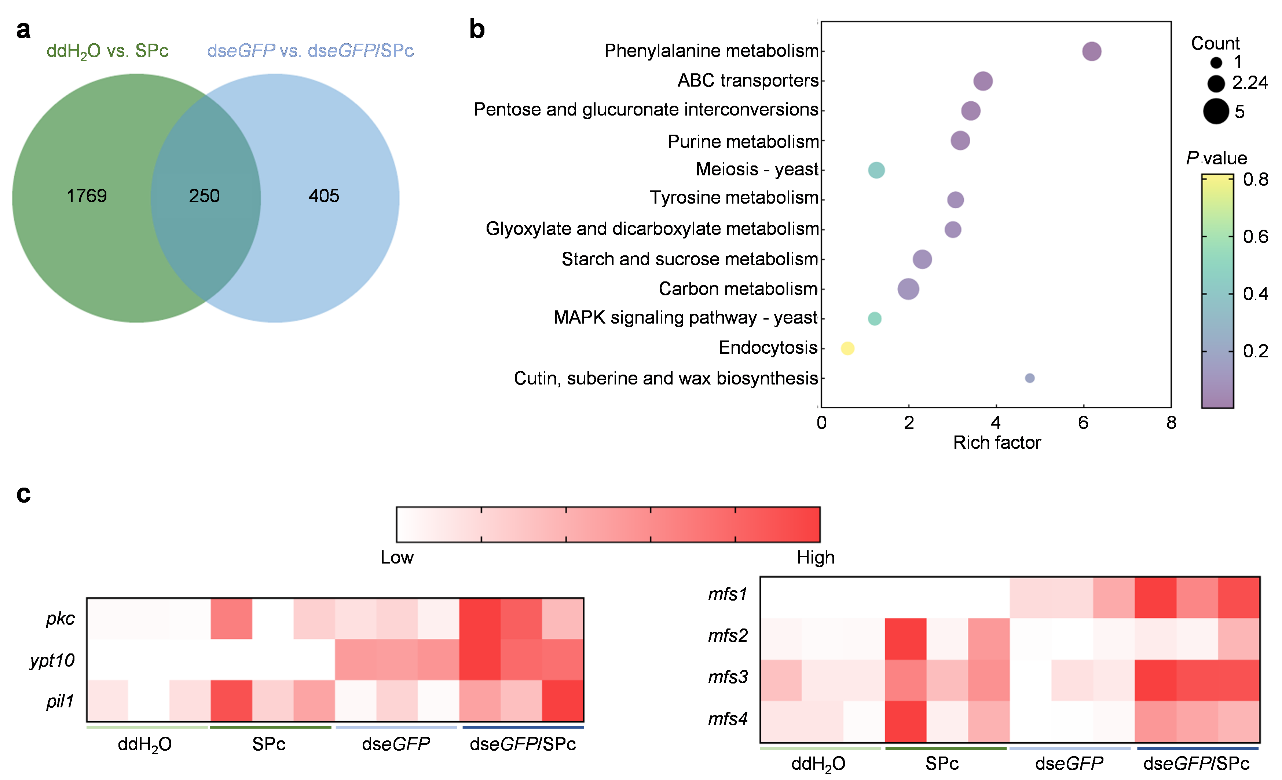


**Figure S4.** **Overlapping DEGs between collected samples for RNA-seq analysis. a** Analysis of DEGs with venn diagram. **b** KEGG enrichment of overlapping DEGs. **c** Heat maps of overlapping DEGs associated with endocytosis and transmembrane transport. Genes with high expression levels are shown in red, while those with low expression levels appear in white.

**
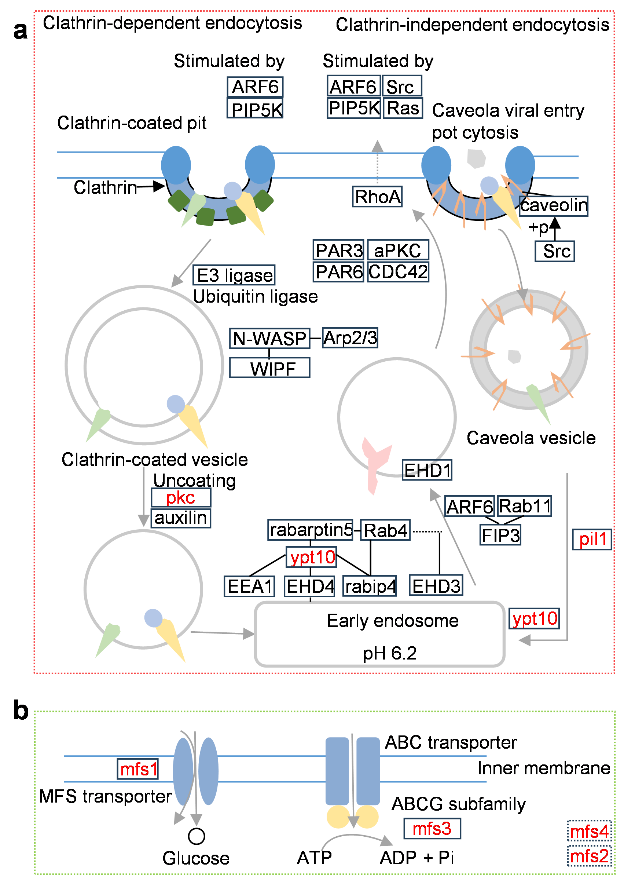
**

**Figure S5.** **Schematic diagram for main transport-related pathways.** **a** Endocytic pathways (Clathrin-dependent and independent endocytosis). Up-regulated genes are shown in red. **b** Transmembrane transport-mediated delivery (MFS transporter and ABC transporter). The *mfs2* and *mfs4* contained MFS domain, but they were not annotated in the KEGG enrichment.

**
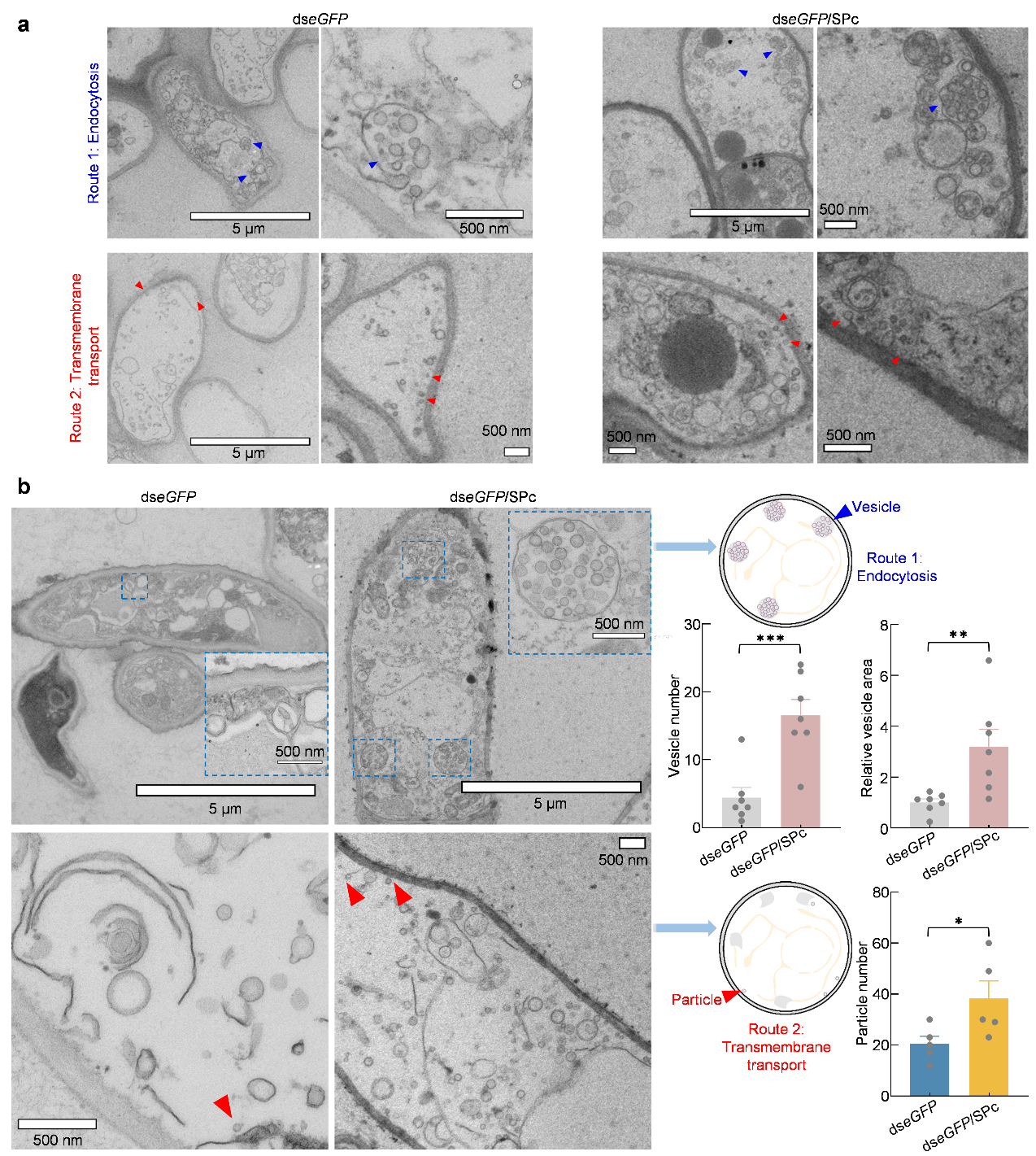
**

**Figure S6.** **Visualization of SPc-mediated dsRNA delivery enhancement via two routes in *B. cinerea*. a** Representative TEM images of ds*eGFP* delivery by SPc via two routes endocytosis and transmembrane transport. **b** SPc-mediated activation of two routes for enhanced dsRNA delivery. In route 1, the number and area of vesicles in the mycelia incubated with ds*eGFP* and ds*eGFP*/SPc complex were quantified. Each treatment was repeated seven times. The asterisk indicates significant difference according to independent *t* test (Two-tailed, ****p* < 0.001, ***p* < 0.01). Vesicle number (*t* = 4.38, df = 12, *p* = 0.0009*)*, and relative vesicle area (*t* = 3.07, df = 12, *p* = 0.0097). In route 2, the particle number in the mycelia incubated with ds*eGFP* and ds*eGFP*/SPc complex was recorded. Each treatment consisted of five independent images. The asterisk indicates significant difference according to independent *t* test (Two-tailed, *t* = 2.34, df = 8, *p* =0.0473). Bar represents mean ± SEM.

**
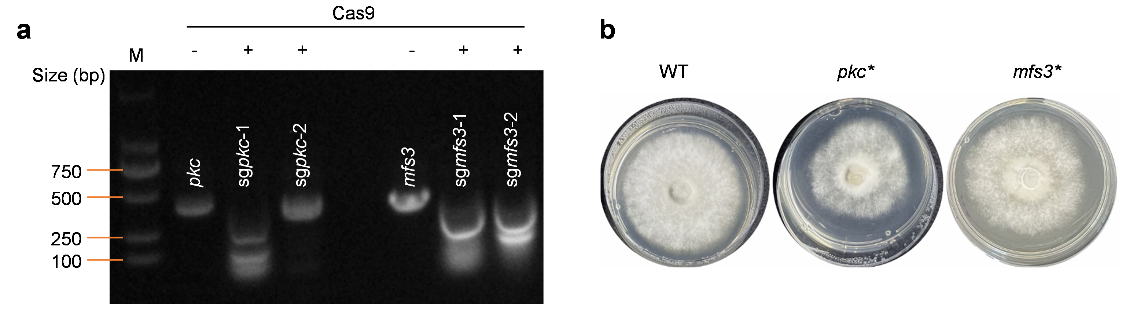
**

**Figure S7.** **sgRNA screening and mutant phenotype. a** sgRNA screening *in vitro*. M: DNA marker. **b** Phenotypes of *pkc* and *mfs3* mutant strains.


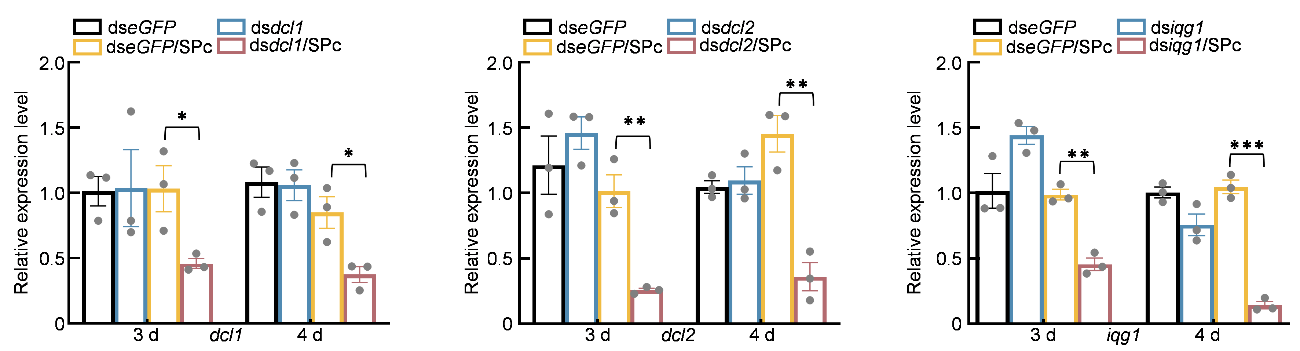


**Figure S8.** **Expression levels of *dcl1*, *dcl2* and *iqg1* in *B. cinerea* incubated with dsRNA/SPc complex using the qRT-PCR.** Each treatment consisted of three replications. The asterisk indicates significant difference according to independent *t* test (Two-tailed, **p* < 0.05, ***p* < 0.01 and ****p* < 0.001). *dcl1* (3 d: *t* = 3.17, df = 4, *p* = 0.0338; 4 d: *t* = 3.51, df = 4, *p* = 0.0247), *dcl2* (3 d: *t* = 6.03, df = 4, *p* = 0.0038; 4 d: *t* =6.20, df = 4, *p* = 0.0034) and *iqg1* (3 d: *t* = 8.56, df = 4, *p* = 0.0010; 4 d: *t* = 15.40, df = 4, *p* = 0.0001). Bar represents mean ± SEM.


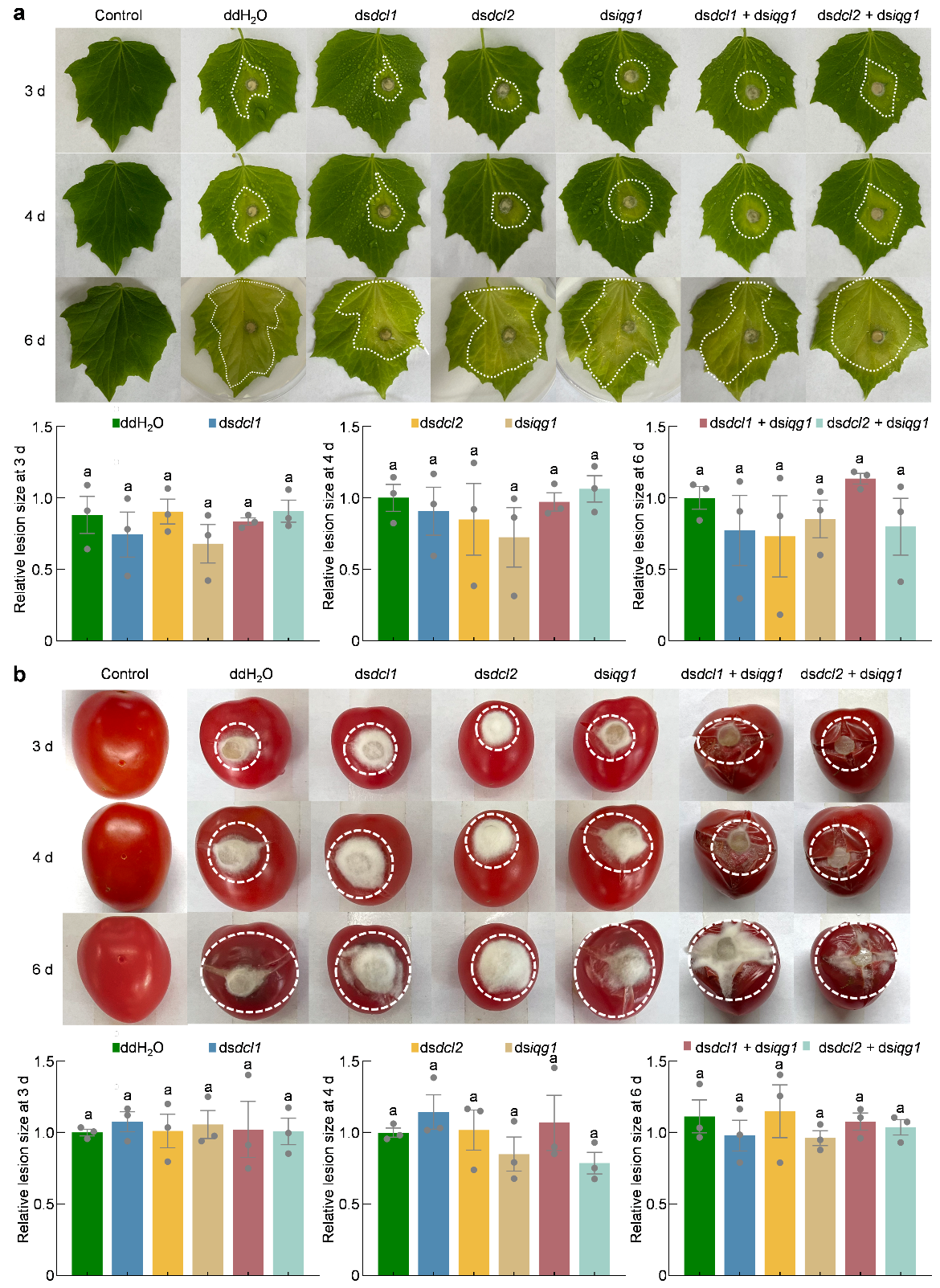


**Figure S9. Protective effects of naked dsRNA toward gray mold disease. a** Protective effects of dsRNA on cucumber leaves. Incisions were made on the leaves, and various formulations were sprayed on the leaves, which were then inoculated with *B. cinerea* mycelium. The images were captured at 3, 4 and 6 d after the inoculation, and the lesion sizes were measured (*n* = three replications). Different letters above each bar indicate significant differences at *p* < 0.05 as determined by one-way ANOVA with Tukey HSD test (3 d: *F_5,12_* = 0.71, *p* = 0.6248; 4 d: *F_5,12_* = 0.56, *p* = 0.7267; 6 d: *F_5,12_* = 0.70, *p* = 0.6331). **b** Protective effects of dsRNA on tomato fruits. Holes were made in the center of fruits, and various formulations were sprayed on the fruits, which were then inoculated with *B. cinerea* mycelium. Images were captured at 3, 4 and 6 d after the inoculation, and the lesion sizes were measured (*n* = three replications). Different letters above each bar indicate significant differences at *p* < 0.05 as determined by one-way ANOVA with Tukey HSD test (3 d: *F_5,12_* = 0.07, *p* = 0.9953; 4 d: *F_5,12_* = 1.18, *p* = 0.3716; 6 d: *F_5,12_* = 0.48, *p* = 0.7829). **a**-**b** Bar represents mean ± SEM*.*

**Table S1. Sequencing quality for RNA-seq analysis.**

| Sample | Clean read | Clean base | GC content | Q30 |
| --- | --- | --- | --- | --- |
| ddH_2_O-1 | 2099342 | 6,283,418,688 | 46.98% | 93.33% |
| ddH_2_O-2 | 20,275,366 | 6,069,758,695 | 46.96% | 92.91% |
| ddH_2_O-3 | 20,753,912 | 6,213,277,867 | 46.92% | 93.40% |
| SPc-1 | 20,802,162 | 6,227,921,280 | 46.94% | 93.10% |
| SPc-2 | 20,650,891 | 6,183,187,828 | 46.95% | 93.64% |
| SPc-3 | 20,416,253 | 6,115,819,126 | 47.03% | 92.87% |
| ds*eGFP*-1 | 21290429 | 6376131144 | 47.09% | 93.23% |
| ds*eGFP*-2 | 24780051 | 7420736294 | 47.06% | 93.84% |
| ds*eGFP*-3 | 25816905 | 7731179880 | 47.03% | 93.31% |
| ds*eGFP*/SPc-1 | 23881947 | 7140905730 | 46.82% | 93.48% |
| ds*eGFP*/SPc-2 | 23613920 | 7071160770 | 46.90% | 93.09% |
| ds*eGFP*/SPc-3 | 26328993 | 7884743874 | 46.96% | 93.42% |

**Table S2. Key DEGs related to endocytosis and transmembrane transport identified from RNA-seq analysis.**

| **Route** | **Gene symbol** | **Abbreviation** |
| --- | --- | --- |
| Endocytosis | *BCIN_13g03260* | *pkc* |
|  | *Bcypt10* | *ypt10* |
|  | *BCIN_11g05390* | *pil1* |
| Transmembrane transport | *BCIN_09g05240* | *mfs1* |
|  | *BCIN_06g01490* | *mfs2* |
|  | *BCIN_06g04860* | *mfs3* |
|  | *BCIN_09g05940* | *mfs4* |

**Table S3. Primers used in this study.**

| **Primer** | **Sequence (5’-3’)** | **Application** |
| --- | --- | --- |
| *eGFP* | TAATACGACTCACTATAGGCACAAGTTCAGCGTGTCCG  TAATACGACTCACTATAGGGTTCACCTTGATGCCGTTC | dsRNA synthesis |
| *tublin* | TAATACGACTCACTATAGGCAACTCTTCCGTCCCGACAA  TAATACGACTCACTATAGGAGAGATCGAGGCATCCTGGT |  |
| *pkc* | TAATACGACTCACTATAGGGGAGCGCTCAAAAGGAGGAT  TAATACGACTCACTATAGGAGTCCGAGAGCATAAAGCCG |  |
| *ypt10* | TAATACGACTCACTATAGGGCCGATCTCGTCGCTAAAGA  TAATACGACTCACTATAGGACCGCGCTCAAAAGTTGTTC |  |
| *pil1* | TAATACGACTCACTATAGGTCCCTCTCTTCCCTTCGTGG  TAATACGACTCACTATAGGAGCACTTTGAGGCTCCTTGG |  |
| *mfs1* | TAATACGACTCACTATAGGAGTGCGGAAATCATCCAAAG  TTAATACGACTCACTATAGGTTTGTCCAGTGGTGACGGTA |  |
| *mfs2* | TAATACGACTCACTATAGGGCATTCGGTTGTGAATTGTG  TAATACGACTCACTATAGGCAAGCCAGATTAGCCTCGAC |  |
| *mfs3* | TAATACGACTCACTATAGGGGTTCTTGGGTATGGCTGAA  TAATACGACTCACTATAGGGCCCAGCCACATCTTGTAAT |  |
| *mfs4* | TAATACGACTCACTATAGGTATCGTCCTTCAATCTGGGC  TAATACGACTCACTATAGGACGAAAGCCAGTCCAGCTAA |  |
| *dcl1* | TTAATACGACTCACTATAGGTGCGGAAGAACTTGAAGGT  TTGCTACA  TAATACGACTCACTATAGGGCAGCAAATGGCATCCGTCCAGATCTGGTCAACACACCAAG |  |
| *dcl2* | TAATACGACTCACTATAGGCTTGGTGTGTTGACCAGATCTGGACGGATGCCATTTGCTGC  TAATACGACTCACTATAGGACTCTTGAGTACTTTCGCCAGCTCAC |  |
| *iqg1* | TAATACGACTCACTATAGGAGGGGAGTAACAGCAGAGCA  TAATACGACTCACTATAGGAGTTCGGCCGTGTGTTTATC |  |
| *q*-*actin* | CTGGAATGTGTAAGGCCGGT  ACCGTGCTCGATTGGGTATC | qRT-PCR |
| *q*-*pkc* | GGGTGTACCTCGCGGATTTT  GGAGCTATCCAAGACGCAGT |  |
| *q*-*ypt10* | GCACCTCCCTCGAAGCTAAG  TCAGAAAACTCGCACCCACA |  |
| *q*-*pil1* | ACACCAATCGTGCCAGGAAA  GCGATTGAACTTCTTCGGCAT |  |
| *q*-*mfs1* | GAGCAAGACCAGCGCTAAGA  CGAGCAGGAGAATGAGCACT |  |
| *q*-*mfs2* | ACCGCAAAGTACGCATGAGA  CACCGCTGCATGATTTGCTT |  |
| *q*-*mfs3* | AATGCGAAGATCGATGGCCT  GGCGAAGGCGTTTCAAAAGA |  |
| *q*-*mfs4* | ACACATCGTTGCCTGGAAGT  AAGTGATCGTTCTGGCGAGG |  |
| *q*-*dcl1* | TGGGCAAAGGCTAGAGCAAA  AGTCTCGAGCTCACCTCCAT |  |
| *q*-*dcl2* | TGCCAAGGCTCTCAGACAAG  CGGAAAAGGGAGGCTTCTGT |  |
| *q*-*iqg1* | TTGGAAGATGCGACCGACTT  TGCCTGGGCTCTGATTGAAG |  |
| sg*pkc*-1-F | AAGCTAATACGACTCACTATAGGAGGAAGCTAATGCCATGGGTTTTAGAGCTAGAAATAGC | Transformation |
| sg*pkc*-2-F | AAGCTAATACGACTCACTATAGATTCGTTCCGATACGTGCGGTTTTAGAGCTAGAAATAGC |  |
| 60 bp-F-*pkc* | TACATATATTCTTACACTTAATTGTACAGTAAGATTTCGTTCATTCAAAAAACCTTTCCAGACTGCCTCAGGTGGGGC |  |
| 60 bp-R-*pkc* | CAAGTTCTAGAATATCCGAATAATAGATCATGGGTGTCATGTAGTTTTGCTCGAAGTTTTTAATGCATTGCAGATGAGCTGTATC |  |
| F-*pkc* | GGATGCCAGCCACGAACT |  |
| R-*pkc* | CAACTCTGACGACACGCAAAG |  |
| sg*mfs3*-1-F | AAGCTAATACGACTCACTATAGAGAGGAAGTAGTTTACGCCGTTTTAGAGCTAGAAATAGC |  |
| sg*mfs3*-2-F | AAGCTAATACGACTCACTATAGGCATGGATCTTCATCATTGGTTTTAGAGCTAGAAATAGC |  |
| sgRNA-scaffold-R | AAAAGCACCGACTCGGTGCCACTTTTTCAAGTTGATAACGGACTAGCCTTATTTTAACTTGCTATTTCTAGCTCTAAAAC |  |
| 60 bp-F-*mfs3* | TAGCACAGAGAAGGAAATCGAAATTCGACTGCATAATTCTATAGTTACACCTGTCTAATCGACTGCCTCAGGTGGGGC |  |
| 60 bp-R-*mfs3* | CAGTAAAAGCGAGTAGAAGCTTGTTAACTCACAGTTTGCATATGACACTTAACGTTGACTTAATGCATTGCAGATGAGCTGTATC |  |
| F-*mfs3* | ATACCTGCATCGACGCAAAG |  |
| R-*mfs3* | AGCCAAGTAAACGGGTCAAA |  |

The T7 promoter sequence is underlined.

**Table S4. Hygromycin resistance sequence used in this study.**

| Pcpc1-HygR-Ttrpc | gactgcctcaggtggggcagtgctagtgtgtgtaccgacccgcaggattggtgctttgcccagagctctacagaatagcgcgcgcatccatatgttagttctgcaattttcttgtatcggtgctgtgactcatacttccccctttggctggccttgcggcaaccaataagaacgcacagtgaaatcttgcgggtggggagtggatccatggcgcctgcattggcttggggacgcgcactgtcgcacacttccatctgacctttcagaagggtttcgtggtgggcaaggaccaaccggttgcgcggccgtgcgtgggtgcctcgcccggcactgccagggccactgcagtggcagtttgctgcctgatacaaaatccttccctccgcccagttttccctctttgaccttcctttcttcttctctgcaaccaaatccaccctatcaaaccaaaacagtatctcgaccgaggtatcaacctgaatcagcaacatcgtagccagcatttgtctccgtctctgcagaaccagcgagttgcaaacattatccaggcaacagggcaccaactcacttcttcggctttcaccaatcggtacagctcttctcagaactcgcgtccgcaacagttctacgcttcctcagcaccttcttcagcttcaatcctgaacactcagaaccgcgcacagcagcgccctcctgttcccttgtttcccaaaagtaccggtagtatttcgcacggaaagcagggcaacaagatgttctcaggtacccatatgaaaaagcctgaactcaccgcgacgtctgtcgagaagtttctgatcgaaaagttcgacagcgtctccgacctgatgcagctctcggagggcgaagaatctcgtgctttcagcttcgatgtaggagggcgtggatatgtcctgcgggtaaatagctgcgccgatggtttctacaaagatcgttatgtttatcggcactttgcatcggccgcgctcccgattccggaagtgcttgacattggggaattcagcgagagcctgacctattgcatctcccgccgtgcacagggtgtcacgttgcaagacctgcctgaaaccgaactgcccgctgttctgcagccggtcgcggaggccatggatgcgatcgctgcggccgatcttagccagacgagcgggttcggcccattcggaccgcaaggaatcggtcaatacactacatggcgtgatttcatatgcgcgattgctgatccccatgtgtatcactggcaaactgtgatggacgacaccgtcagtgcgtccgtcgcgcaggctctcgatgagctgatgctttgggccgaggactgccccgaagtccggcacctcgtgcacgcggatttcggctccaacaatgtcctgacggacaatggccgcataacagcggtcattgactggagcgaggcgatgttcggggattcccaatacgaggtcgccaacatcttcttctggaggccgtggttggcttgtatggagcagcagacgcgctacttcgagcggaggcatccggagcttgcaggatcgccgcgcctccgggcgtatatgctccgcattggtcttgaccaactctatcagagcttggttgacggcaatttcgatgatgcagcttgggcgcagggtcgatgcgacgcaatcgtccgatccggagccgggactgtcgggcgtacacaaatcgcccgcagaagcgcggccgtctggaccgatggctgtgtagaagtactcgccgatagtggaaaccgacgccccagcactcgtccgagggcaaaggaatagagtagatgccgaccgggatccacttaacgttactgaaatcatcaaacagcttgacgaatctggatataagatcgttggtgtcgatgtcagctccggagttgagacaaatggtgttcaggatctcgataagatacgttcatttgtccaagcagcaaagagtgccttctagtgatttaatagctccatgtcaacaagaataaaacgcgtttcgggtttacctcttccagatacagctcatctgcaatgcatta |
| --- | --- |

The sequences of Pcpc1, HygR and Ttrpc are shown in blue, green and red, respectively.
