# Supplementary figures and images for: Interactional Endocytosis and Transmembrane Transport Promote Cellular Internalization of Nano‐Delivered RNA Drugs for Efficient Control of Crop Diseases
Source: Plant Biotechnol J. 2026 May 29:10.1111/pbi.70689. Online ahead of print. doi: 10.1111/pbi.70689 (PMC13398849; doi:10.1111/pbi.70689)

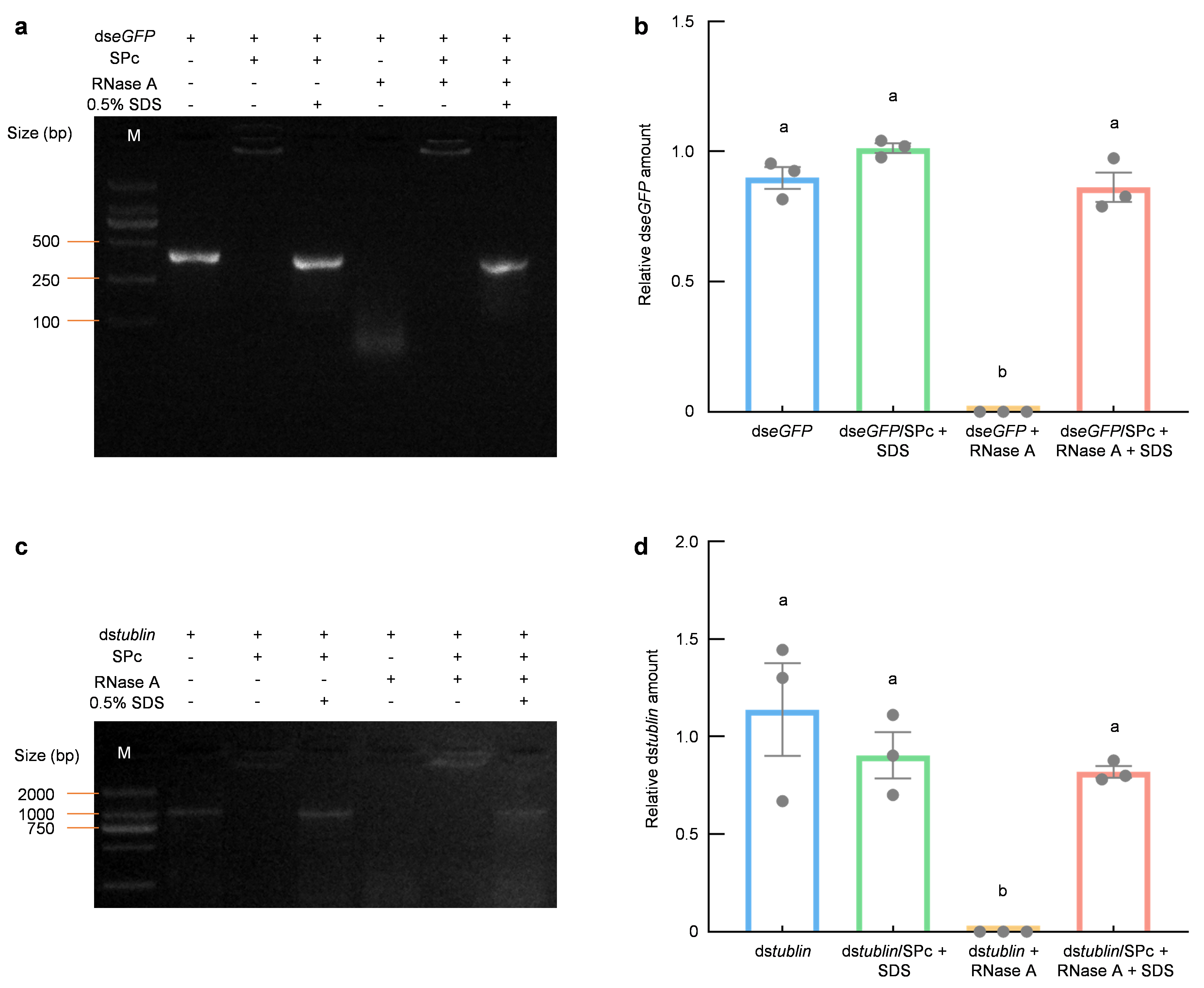

Supplement: Supplementary file 1 — Figure S1: Enhanced stability of SPc‐loaded dsRNA treated with RNase A. (a) Complexation with SPc prevented the dseGFP migration to the positive electrode. To assess the protective effect of SPc on dseGFP (420 bp), electrophoresis was conducted after the incubation of RNase A with naked dseGFP or dseGFP/SPc complex for 20 min. The 0.5% SDS was added to release dseGFP from the dseGFP/SPc complex. (b) Relative dseGFP amount was analysed using the Image J 1.8 software (n = 3 replications). Different letters above each bar indicate significant differences at p < 0.05 as determined by one‐way ANOVA with Tukey HSD test (F 3,8 = 166.3, p < 0.0001). (c) Complexation with SPc prevented the dstublin migration to the positive electrode. To assess the protective effect of SPc on dstublin (1047 bp), electrophoresis was conducted after the incubation of RNase A with naked dstublin or dstublin/SPc complex for 20 min. The 0.5% SDS was added to release dstublin from the dstublin/SPc complex. (d) Relative dstublin amount was analysed using the Image J 1.8 software (n = 3 replications). Different letters above each bar indicate significant differences at p < 0.05 as determined by one‐way ANOVA with Tukey HSD test (F 3,8 = 13.76, p = 0.0016). Bar represents mean ± SEM. Figure S2: Standard curve for quantifying dseGFP using the qRT‐PCR. The Ct numbers of naked dseGFP with various qualities were determined. Figure S3: Pearson correlation between collected samples for RNA‐seq analysis. Figure S4: Overlapping DEGs between collected samples for RNA‐seq analysis. (a) Analysis of DEGs with Venn diagram. (b) KEGG enrichment of overlapping DEGs. (c) Heat maps of overlapping DEGs associated with endocytosis and transmembrane transport. Genes with high expression levels are shown in red, while those with low expression levels appear in white. Figure S5: Schematic diagram for main transport‐related pathways. (a) Endocytic pathways (Clathrin‐dependent and independent endocytosis). Up‐regulated gene [file PBI-9999-0-s001.zip › 1_FigureS1.tif]

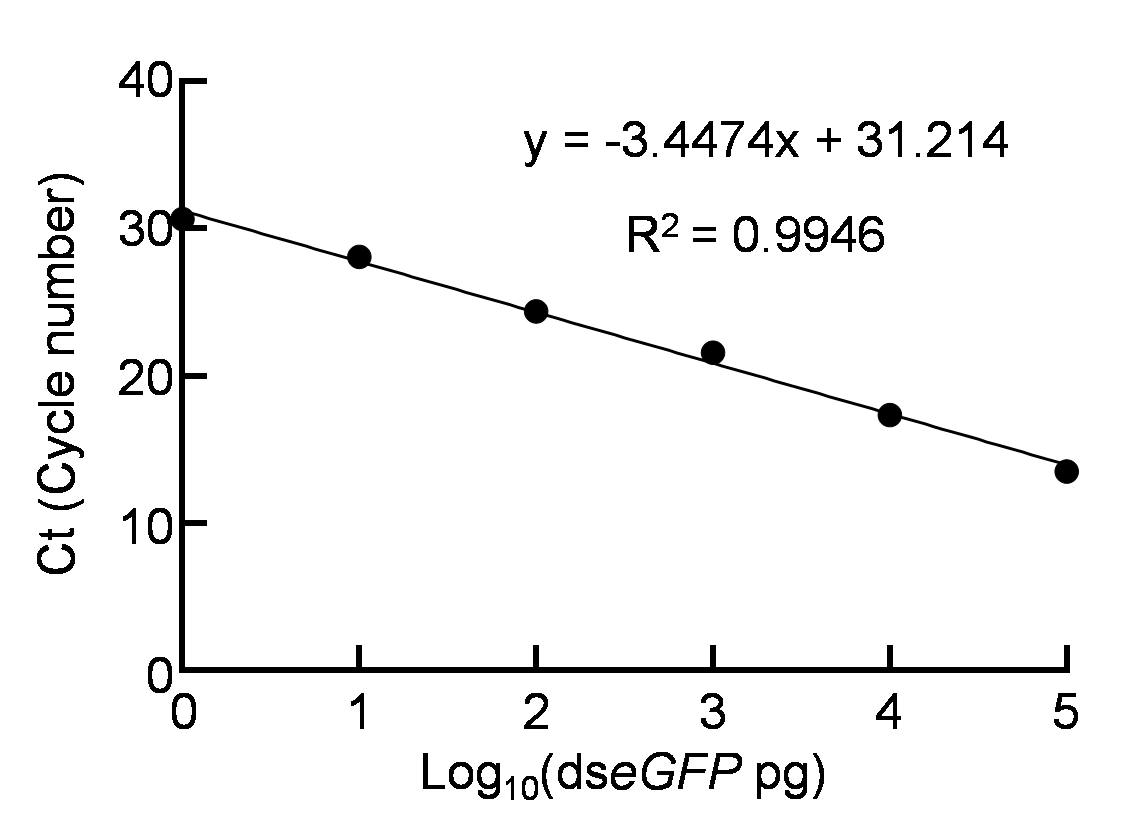

Supplement: Supplementary file 1 — Figure S1: Enhanced stability of SPc‐loaded dsRNA treated with RNase A. (a) Complexation with SPc prevented the dseGFP migration to the positive electrode. To assess the protective effect of SPc on dseGFP (420 bp), electrophoresis was conducted after the incubation of RNase A with naked dseGFP or dseGFP/SPc complex for 20 min. The 0.5% SDS was added to release dseGFP from the dseGFP/SPc complex. (b) Relative dseGFP amount was analysed using the Image J 1.8 software (n = 3 replications). Different letters above each bar indicate significant differences at p < 0.05 as determined by one‐way ANOVA with Tukey HSD test (F 3,8 = 166.3, p < 0.0001). (c) Complexation with SPc prevented the dstublin migration to the positive electrode. To assess the protective effect of SPc on dstublin (1047 bp), electrophoresis was conducted after the incubation of RNase A with naked dstublin or dstublin/SPc complex for 20 min. The 0.5% SDS was added to release dstublin from the dstublin/SPc complex. (d) Relative dstublin amount was analysed using the Image J 1.8 software (n = 3 replications). Different letters above each bar indicate significant differences at p < 0.05 as determined by one‐way ANOVA with Tukey HSD test (F 3,8 = 13.76, p = 0.0016). Bar represents mean ± SEM. Figure S2: Standard curve for quantifying dseGFP using the qRT‐PCR. The Ct numbers of naked dseGFP with various qualities were determined. Figure S3: Pearson correlation between collected samples for RNA‐seq analysis. Figure S4: Overlapping DEGs between collected samples for RNA‐seq analysis. (a) Analysis of DEGs with Venn diagram. (b) KEGG enrichment of overlapping DEGs. (c) Heat maps of overlapping DEGs associated with endocytosis and transmembrane transport. Genes with high expression levels are shown in red, while those with low expression levels appear in white. Figure S5: Schematic diagram for main transport‐related pathways. (a) Endocytic pathways (Clathrin‐dependent and independent endocytosis). Up‐regulated gene [file PBI-9999-0-s001.zip › 1_FigureS2.tif]

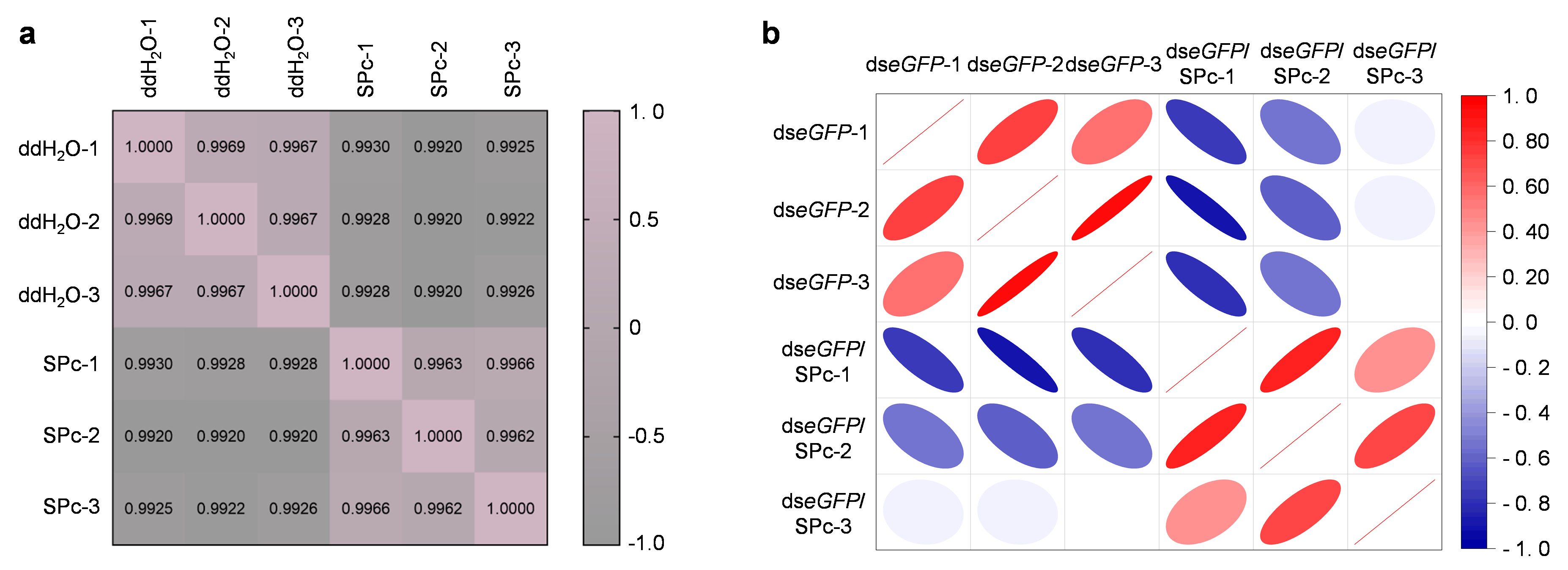

Supplement: Supplementary file 1 — Figure S1: Enhanced stability of SPc‐loaded dsRNA treated with RNase A. (a) Complexation with SPc prevented the dseGFP migration to the positive electrode. To assess the protective effect of SPc on dseGFP (420 bp), electrophoresis was conducted after the incubation of RNase A with naked dseGFP or dseGFP/SPc complex for 20 min. The 0.5% SDS was added to release dseGFP from the dseGFP/SPc complex. (b) Relative dseGFP amount was analysed using the Image J 1.8 software (n = 3 replications). Different letters above each bar indicate significant differences at p < 0.05 as determined by one‐way ANOVA with Tukey HSD test (F 3,8 = 166.3, p < 0.0001). (c) Complexation with SPc prevented the dstublin migration to the positive electrode. To assess the protective effect of SPc on dstublin (1047 bp), electrophoresis was conducted after the incubation of RNase A with naked dstublin or dstublin/SPc complex for 20 min. The 0.5% SDS was added to release dstublin from the dstublin/SPc complex. (d) Relative dstublin amount was analysed using the Image J 1.8 software (n = 3 replications). Different letters above each bar indicate significant differences at p < 0.05 as determined by one‐way ANOVA with Tukey HSD test (F 3,8 = 13.76, p = 0.0016). Bar represents mean ± SEM. Figure S2: Standard curve for quantifying dseGFP using the qRT‐PCR. The Ct numbers of naked dseGFP with various qualities were determined. Figure S3: Pearson correlation between collected samples for RNA‐seq analysis. Figure S4: Overlapping DEGs between collected samples for RNA‐seq analysis. (a) Analysis of DEGs with Venn diagram. (b) KEGG enrichment of overlapping DEGs. (c) Heat maps of overlapping DEGs associated with endocytosis and transmembrane transport. Genes with high expression levels are shown in red, while those with low expression levels appear in white. Figure S5: Schematic diagram for main transport‐related pathways. (a) Endocytic pathways (Clathrin‐dependent and independent endocytosis). Up‐regulated gene [file PBI-9999-0-s001.zip › 1_FigureS3.tif]

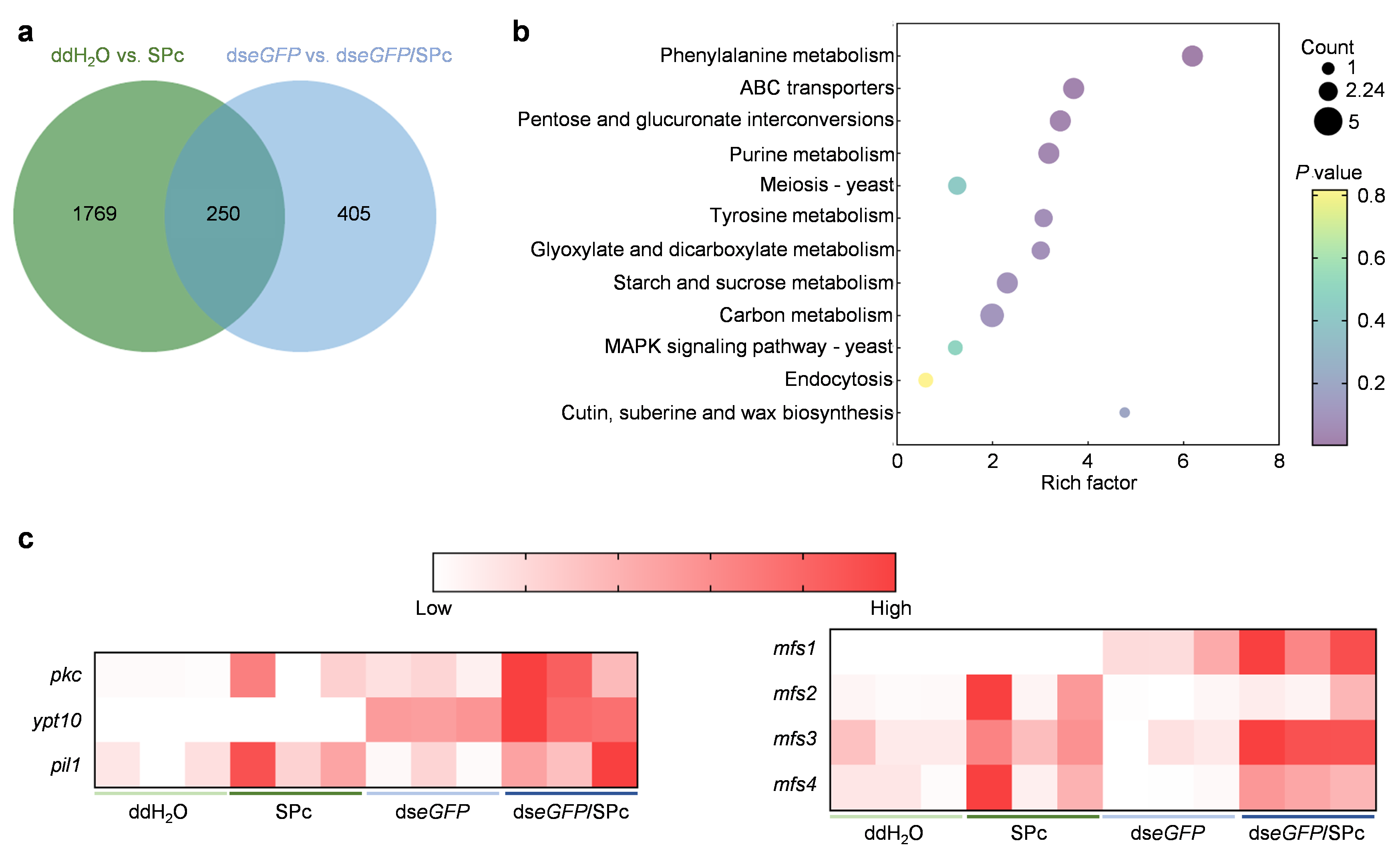

Supplement: Supplementary file 1 — Figure S1: Enhanced stability of SPc‐loaded dsRNA treated with RNase A. (a) Complexation with SPc prevented the dseGFP migration to the positive electrode. To assess the protective effect of SPc on dseGFP (420 bp), electrophoresis was conducted after the incubation of RNase A with naked dseGFP or dseGFP/SPc complex for 20 min. The 0.5% SDS was added to release dseGFP from the dseGFP/SPc complex. (b) Relative dseGFP amount was analysed using the Image J 1.8 software (n = 3 replications). Different letters above each bar indicate significant differences at p < 0.05 as determined by one‐way ANOVA with Tukey HSD test (F 3,8 = 166.3, p < 0.0001). (c) Complexation with SPc prevented the dstublin migration to the positive electrode. To assess the protective effect of SPc on dstublin (1047 bp), electrophoresis was conducted after the incubation of RNase A with naked dstublin or dstublin/SPc complex for 20 min. The 0.5% SDS was added to release dstublin from the dstublin/SPc complex. (d) Relative dstublin amount was analysed using the Image J 1.8 software (n = 3 replications). Different letters above each bar indicate significant differences at p < 0.05 as determined by one‐way ANOVA with Tukey HSD test (F 3,8 = 13.76, p = 0.0016). Bar represents mean ± SEM. Figure S2: Standard curve for quantifying dseGFP using the qRT‐PCR. The Ct numbers of naked dseGFP with various qualities were determined. Figure S3: Pearson correlation between collected samples for RNA‐seq analysis. Figure S4: Overlapping DEGs between collected samples for RNA‐seq analysis. (a) Analysis of DEGs with Venn diagram. (b) KEGG enrichment of overlapping DEGs. (c) Heat maps of overlapping DEGs associated with endocytosis and transmembrane transport. Genes with high expression levels are shown in red, while those with low expression levels appear in white. Figure S5: Schematic diagram for main transport‐related pathways. (a) Endocytic pathways (Clathrin‐dependent and independent endocytosis). Up‐regulated gene [file PBI-9999-0-s001.zip › 1_FigureS4.tif]

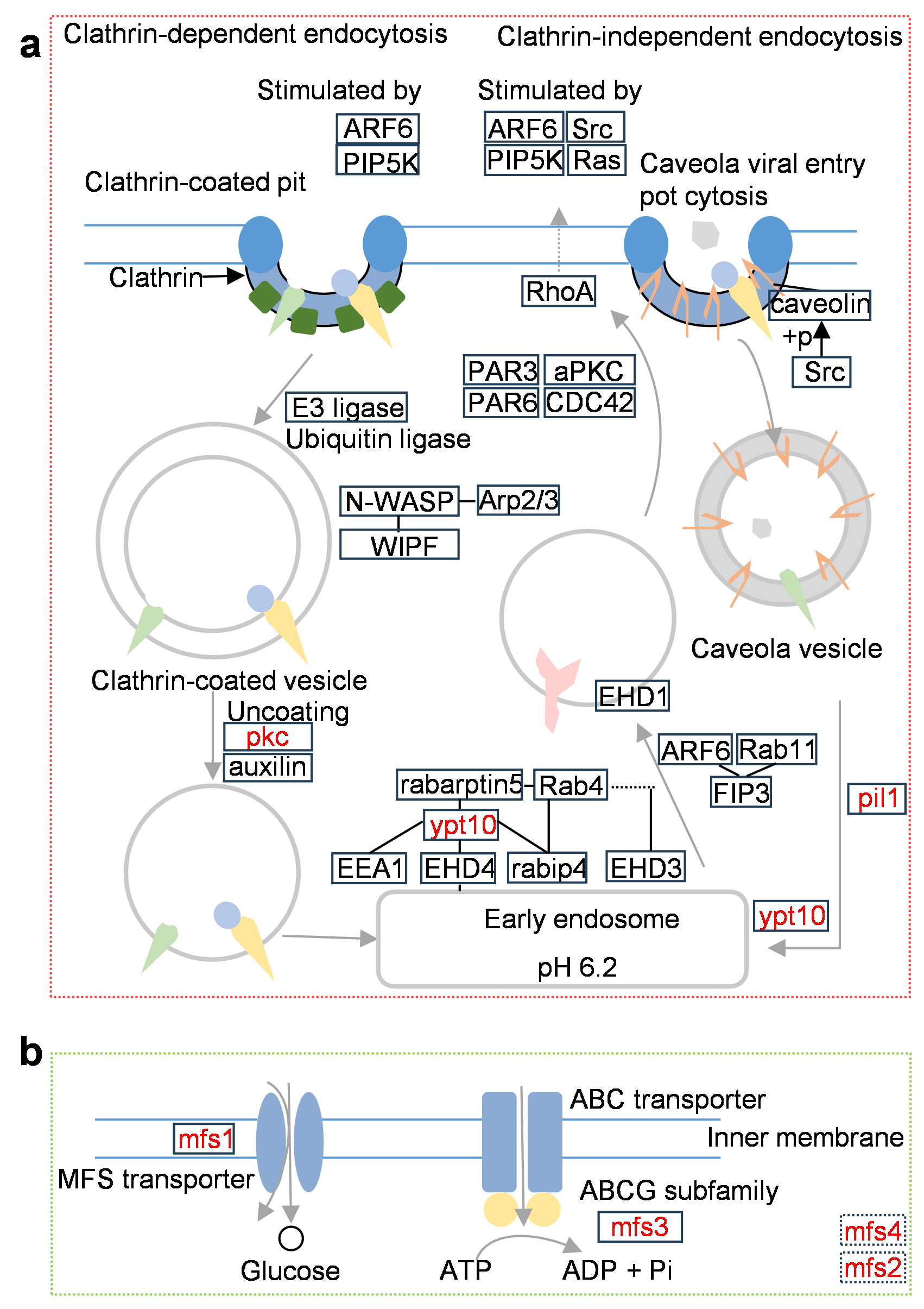

Supplement: Supplementary file 1 — Figure S1: Enhanced stability of SPc‐loaded dsRNA treated with RNase A. (a) Complexation with SPc prevented the dseGFP migration to the positive electrode. To assess the protective effect of SPc on dseGFP (420 bp), electrophoresis was conducted after the incubation of RNase A with naked dseGFP or dseGFP/SPc complex for 20 min. The 0.5% SDS was added to release dseGFP from the dseGFP/SPc complex. (b) Relative dseGFP amount was analysed using the Image J 1.8 software (n = 3 replications). Different letters above each bar indicate significant differences at p < 0.05 as determined by one‐way ANOVA with Tukey HSD test (F 3,8 = 166.3, p < 0.0001). (c) Complexation with SPc prevented the dstublin migration to the positive electrode. To assess the protective effect of SPc on dstublin (1047 bp), electrophoresis was conducted after the incubation of RNase A with naked dstublin or dstublin/SPc complex for 20 min. The 0.5% SDS was added to release dstublin from the dstublin/SPc complex. (d) Relative dstublin amount was analysed using the Image J 1.8 software (n = 3 replications). Different letters above each bar indicate significant differences at p < 0.05 as determined by one‐way ANOVA with Tukey HSD test (F 3,8 = 13.76, p = 0.0016). Bar represents mean ± SEM. Figure S2: Standard curve for quantifying dseGFP using the qRT‐PCR. The Ct numbers of naked dseGFP with various qualities were determined. Figure S3: Pearson correlation between collected samples for RNA‐seq analysis. Figure S4: Overlapping DEGs between collected samples for RNA‐seq analysis. (a) Analysis of DEGs with Venn diagram. (b) KEGG enrichment of overlapping DEGs. (c) Heat maps of overlapping DEGs associated with endocytosis and transmembrane transport. Genes with high expression levels are shown in red, while those with low expression levels appear in white. Figure S5: Schematic diagram for main transport‐related pathways. (a) Endocytic pathways (Clathrin‐dependent and independent endocytosis). Up‐regulated gene [file PBI-9999-0-s001.zip › 1_FigureS5.tif]

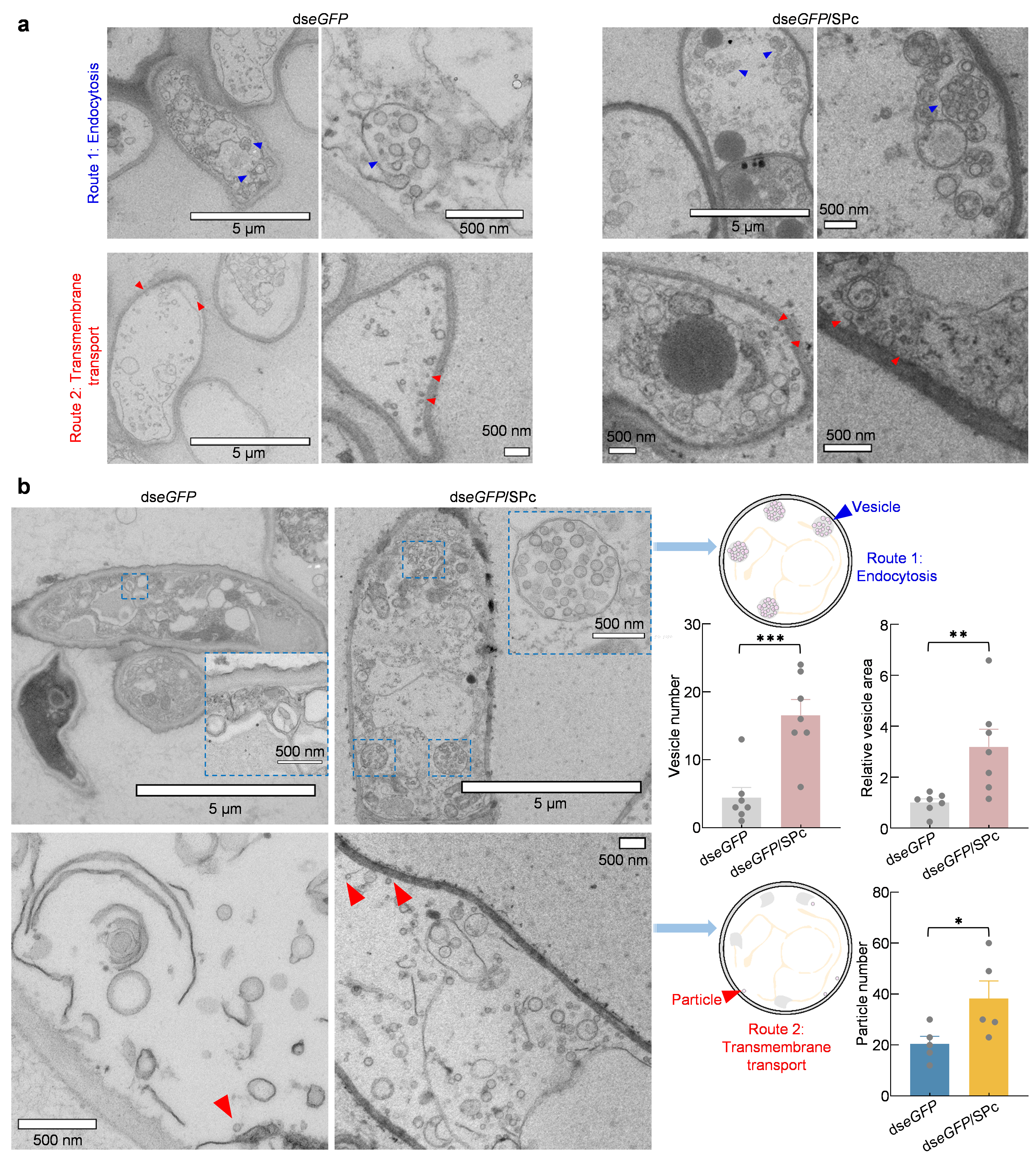

Supplement: Supplementary file 1 — Figure S1: Enhanced stability of SPc‐loaded dsRNA treated with RNase A. (a) Complexation with SPc prevented the dseGFP migration to the positive electrode. To assess the protective effect of SPc on dseGFP (420 bp), electrophoresis was conducted after the incubation of RNase A with naked dseGFP or dseGFP/SPc complex for 20 min. The 0.5% SDS was added to release dseGFP from the dseGFP/SPc complex. (b) Relative dseGFP amount was analysed using the Image J 1.8 software (n = 3 replications). Different letters above each bar indicate significant differences at p < 0.05 as determined by one‐way ANOVA with Tukey HSD test (F 3,8 = 166.3, p < 0.0001). (c) Complexation with SPc prevented the dstublin migration to the positive electrode. To assess the protective effect of SPc on dstublin (1047 bp), electrophoresis was conducted after the incubation of RNase A with naked dstublin or dstublin/SPc complex for 20 min. The 0.5% SDS was added to release dstublin from the dstublin/SPc complex. (d) Relative dstublin amount was analysed using the Image J 1.8 software (n = 3 replications). Different letters above each bar indicate significant differences at p < 0.05 as determined by one‐way ANOVA with Tukey HSD test (F 3,8 = 13.76, p = 0.0016). Bar represents mean ± SEM. Figure S2: Standard curve for quantifying dseGFP using the qRT‐PCR. The Ct numbers of naked dseGFP with various qualities were determined. Figure S3: Pearson correlation between collected samples for RNA‐seq analysis. Figure S4: Overlapping DEGs between collected samples for RNA‐seq analysis. (a) Analysis of DEGs with Venn diagram. (b) KEGG enrichment of overlapping DEGs. (c) Heat maps of overlapping DEGs associated with endocytosis and transmembrane transport. Genes with high expression levels are shown in red, while those with low expression levels appear in white. Figure S5: Schematic diagram for main transport‐related pathways. (a) Endocytic pathways (Clathrin‐dependent and independent endocytosis). Up‐regulated gene [file PBI-9999-0-s001.zip › 1_FigureS6.tif]

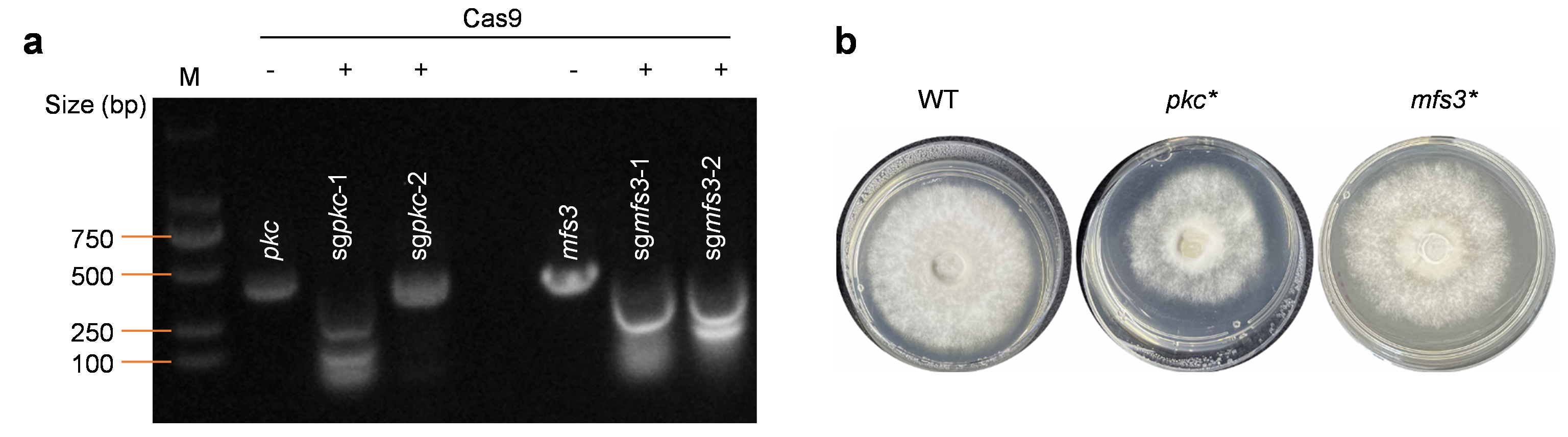

Supplement: Supplementary file 1 — Figure S1: Enhanced stability of SPc‐loaded dsRNA treated with RNase A. (a) Complexation with SPc prevented the dseGFP migration to the positive electrode. To assess the protective effect of SPc on dseGFP (420 bp), electrophoresis was conducted after the incubation of RNase A with naked dseGFP or dseGFP/SPc complex for 20 min. The 0.5% SDS was added to release dseGFP from the dseGFP/SPc complex. (b) Relative dseGFP amount was analysed using the Image J 1.8 software (n = 3 replications). Different letters above each bar indicate significant differences at p < 0.05 as determined by one‐way ANOVA with Tukey HSD test (F 3,8 = 166.3, p < 0.0001). (c) Complexation with SPc prevented the dstublin migration to the positive electrode. To assess the protective effect of SPc on dstublin (1047 bp), electrophoresis was conducted after the incubation of RNase A with naked dstublin or dstublin/SPc complex for 20 min. The 0.5% SDS was added to release dstublin from the dstublin/SPc complex. (d) Relative dstublin amount was analysed using the Image J 1.8 software (n = 3 replications). Different letters above each bar indicate significant differences at p < 0.05 as determined by one‐way ANOVA with Tukey HSD test (F 3,8 = 13.76, p = 0.0016). Bar represents mean ± SEM. Figure S2: Standard curve for quantifying dseGFP using the qRT‐PCR. The Ct numbers of naked dseGFP with various qualities were determined. Figure S3: Pearson correlation between collected samples for RNA‐seq analysis. Figure S4: Overlapping DEGs between collected samples for RNA‐seq analysis. (a) Analysis of DEGs with Venn diagram. (b) KEGG enrichment of overlapping DEGs. (c) Heat maps of overlapping DEGs associated with endocytosis and transmembrane transport. Genes with high expression levels are shown in red, while those with low expression levels appear in white. Figure S5: Schematic diagram for main transport‐related pathways. (a) Endocytic pathways (Clathrin‐dependent and independent endocytosis). Up‐regulated gene [file PBI-9999-0-s001.zip › 1_FigureS7.tif]

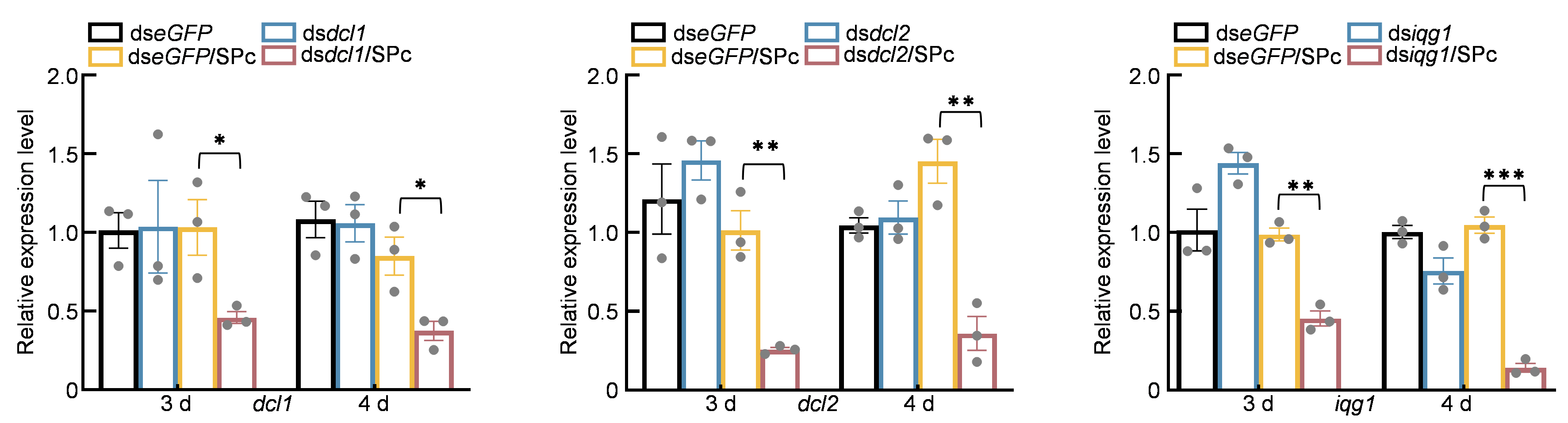

Supplement: Supplementary file 1 — Figure S1: Enhanced stability of SPc‐loaded dsRNA treated with RNase A. (a) Complexation with SPc prevented the dseGFP migration to the positive electrode. To assess the protective effect of SPc on dseGFP (420 bp), electrophoresis was conducted after the incubation of RNase A with naked dseGFP or dseGFP/SPc complex for 20 min. The 0.5% SDS was added to release dseGFP from the dseGFP/SPc complex. (b) Relative dseGFP amount was analysed using the Image J 1.8 software (n = 3 replications). Different letters above each bar indicate significant differences at p < 0.05 as determined by one‐way ANOVA with Tukey HSD test (F 3,8 = 166.3, p < 0.0001). (c) Complexation with SPc prevented the dstublin migration to the positive electrode. To assess the protective effect of SPc on dstublin (1047 bp), electrophoresis was conducted after the incubation of RNase A with naked dstublin or dstublin/SPc complex for 20 min. The 0.5% SDS was added to release dstublin from the dstublin/SPc complex. (d) Relative dstublin amount was analysed using the Image J 1.8 software (n = 3 replications). Different letters above each bar indicate significant differences at p < 0.05 as determined by one‐way ANOVA with Tukey HSD test (F 3,8 = 13.76, p = 0.0016). Bar represents mean ± SEM. Figure S2: Standard curve for quantifying dseGFP using the qRT‐PCR. The Ct numbers of naked dseGFP with various qualities were determined. Figure S3: Pearson correlation between collected samples for RNA‐seq analysis. Figure S4: Overlapping DEGs between collected samples for RNA‐seq analysis. (a) Analysis of DEGs with Venn diagram. (b) KEGG enrichment of overlapping DEGs. (c) Heat maps of overlapping DEGs associated with endocytosis and transmembrane transport. Genes with high expression levels are shown in red, while those with low expression levels appear in white. Figure S5: Schematic diagram for main transport‐related pathways. (a) Endocytic pathways (Clathrin‐dependent and independent endocytosis). Up‐regulated gene [file PBI-9999-0-s001.zip › 1_FigureS8.tif]

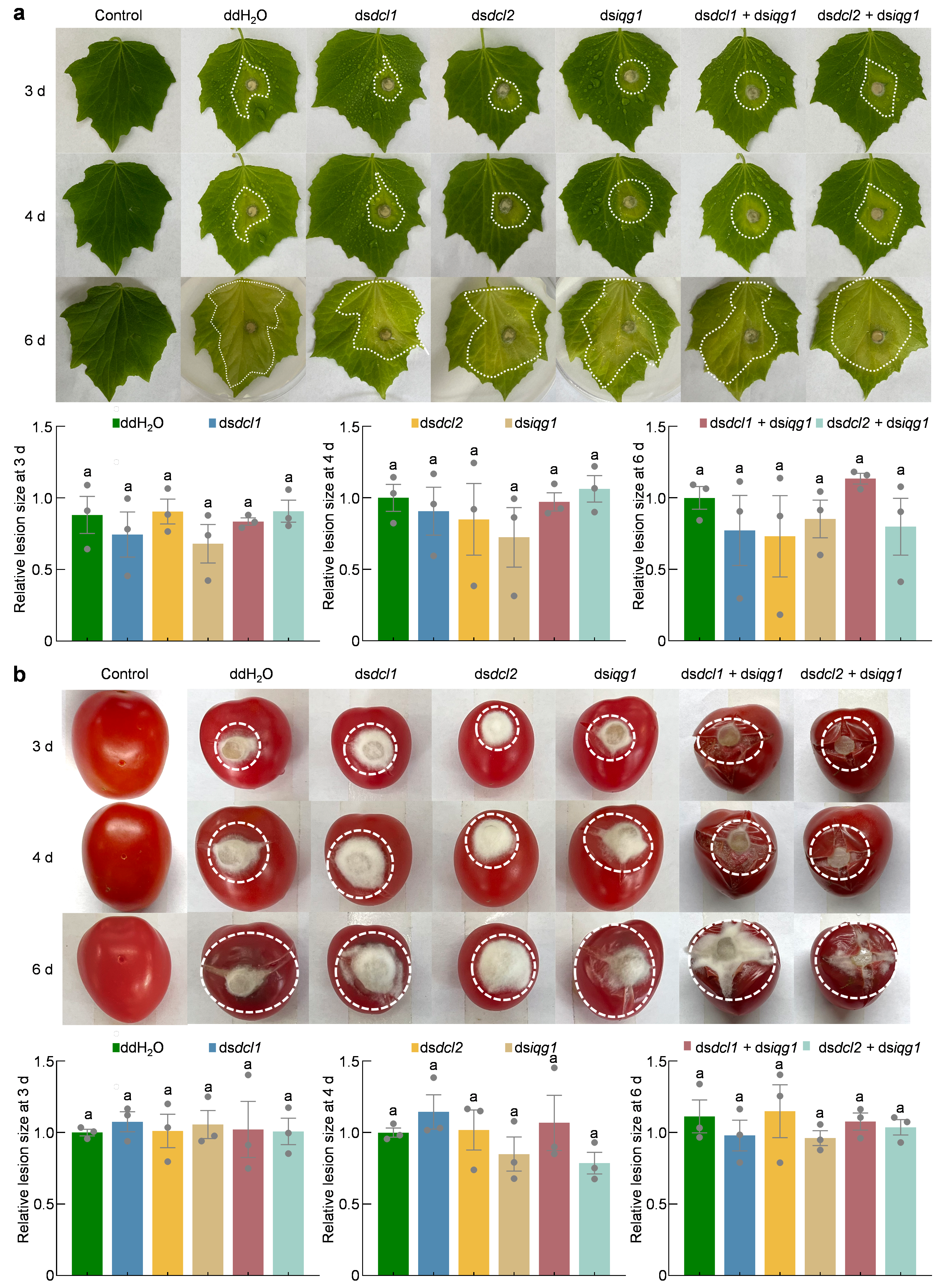

Supplement: Supplementary file 1 — Figure S1: Enhanced stability of SPc‐loaded dsRNA treated with RNase A. (a) Complexation with SPc prevented the dseGFP migration to the positive electrode. To assess the protective effect of SPc on dseGFP (420 bp), electrophoresis was conducted after the incubation of RNase A with naked dseGFP or dseGFP/SPc complex for 20 min. The 0.5% SDS was added to release dseGFP from the dseGFP/SPc complex. (b) Relative dseGFP amount was analysed using the Image J 1.8 software (n = 3 replications). Different letters above each bar indicate significant differences at p < 0.05 as determined by one‐way ANOVA with Tukey HSD test (F 3,8 = 166.3, p < 0.0001). (c) Complexation with SPc prevented the dstublin migration to the positive electrode. To assess the protective effect of SPc on dstublin (1047 bp), electrophoresis was conducted after the incubation of RNase A with naked dstublin or dstublin/SPc complex for 20 min. The 0.5% SDS was added to release dstublin from the dstublin/SPc complex. (d) Relative dstublin amount was analysed using the Image J 1.8 software (n = 3 replications). Different letters above each bar indicate significant differences at p < 0.05 as determined by one‐way ANOVA with Tukey HSD test (F 3,8 = 13.76, p = 0.0016). Bar represents mean ± SEM. Figure S2: Standard curve for quantifying dseGFP using the qRT‐PCR. The Ct numbers of naked dseGFP with various qualities were determined. Figure S3: Pearson correlation between collected samples for RNA‐seq analysis. Figure S4: Overlapping DEGs between collected samples for RNA‐seq analysis. (a) Analysis of DEGs with Venn diagram. (b) KEGG enrichment of overlapping DEGs. (c) Heat maps of overlapping DEGs associated with endocytosis and transmembrane transport. Genes with high expression levels are shown in red, while those with low expression levels appear in white. Figure S5: Schematic diagram for main transport‐related pathways. (a) Endocytic pathways (Clathrin‐dependent and independent endocytosis). Up‐regulated gene [file PBI-9999-0-s001.zip › 1_FigureS9.tif]
